# Galectin-1 activates carbonic anhydrase IX and modulates glioma metabolism

**DOI:** 10.1038/s41419-022-05024-z

**Published:** 2022-06-30

**Authors:** Maheedhara R. Guda, Andrew J. Tsung, Swapna Asuthkar, Kiran K. Velpula

**Affiliations:** 1 Department of Cancer Biology and Pharmacology, University of Illinois College of Medicine at Peoria, Peoria, IL USA; 2 Department of Neurosurgery, University of Illinois College of Medicine at Peoria, Peoria, IL USA; 3 Illinois Neurological Institute, Peoria, IL USA; 4 Department of Pediatrics, University of Illinois College of Medicine at Peoria, Peoria, IL USA

**Keywords:** Cancer metabolism, Cell signalling

## Abstract

Galectins are a family of β-galactose-specific binding proteins residing within the cytosol or nucleus, with a highly conserved carbohydrate recognition domain across many species. Accumulating evidence shows that Galectin 1 (Gal-1) plays an essential role in cancer, and its expression correlates with tumor aggressiveness and progression. Our preliminary data showed Gal-1 promotes glioma stem cell (GSC) growth via increased Warburg effect. mRNA expression and clinical data were obtained from The Cancer Genome Atlas database. The immunoblot analysis conducted using our cohort of human glioblastoma patient specimens (hGBM), confirmed Gal-1 upregulation in GBM. GC/MS analysis to evaluate the effects of Gal-1 depletion showed elevated levels of α-ketoglutaric acid, and citric acid with a concomitant reduction in lactic acid levels. Using Biolog microplate-1 mitochondrial functional assay, we confirmed that the depletion of Gal-1 increases the expression levels of the enzymes from the TCA cycle, suggesting a reversal of the Warburg phenotype. Manipulation of Gal-1 using RNA interference showed reduced ATP, lactate levels, cell viability, colony-forming abilities, and increased expression levels of genes implicated in the induction of apoptosis. Gal-1 exerts its metabolic role via regulating the expression of carbonic anhydrase IX (CA-IX), a surrogate marker for hypoxia. CA-IX functions downstream to Gal-1, and co-immunoprecipitation experiments along with proximity ligation assays confirm that Gal-1 physically associates with CA-IX to regulate its expression. Further, silencing of Gal-1 in mice models showed reduced tumor burden and increased survival compared to the mice implanted with GSC controls. Further investigation of Gal-1 in GSC progression and metabolic reprogramming is warranted.

## Introduction

Glioblastoma (GBM) represents over half of all central nervous system tumors and is the most common primary brain tumor in adults [1–3]. GBM is portrayed by a dismal prognosis, and patients with GBM show a median survival of 12–20 months [4–7], despite the current standard of care that includes maximum surgical resection, followed by chemotherapy and radiotherapy [8]. GBM tumors are significantly heterogeneous involving cancer stem cells. Cancer stem cells (CSCs) derived from GBM are alluded to as glioma stem cells (GSC) [9, 10]. These GSCs show self-renewal capacity, recurrence, resistance to chemotherapy or radiotherapy, and the ability to form tumors in mice, contributing to overall poor prognosis [11–14]. GSCs mimic the human GBM tumor phenotype, and hence, understanding metabolic factors in GSC proliferation may provide new avenues of treatment in GBM [15].

Galectins are a family of mammalian beta-galactoside-binding proteins present in the cytosol and nucleus [16]. Approximately 15 galectins have been identified and share a characteristic carbohydrate recognition domain. Among these, Galectin 1 (Gal-1) and Galectin 3 (Gal-3) have been studied in relation to GBMs. The Gal-1, a lactose binding lectin, is encoded by the *LGALS1* gene, and is characterized by its ability to bind intact β-galactose molecules on the cell surface and extracellular matrix [17]. Accumulating evidence shows that Gal-1 plays an important role in cancer since its expression correlates with tumor aggressiveness and progression [18–20]. Increased expression of Gal-1 promotes cancer cell proliferation [21], cell cycle [22, 23], angiogenesis [24, 25], invasion [26], migration [18, 27], and metabolism [28] in colon, breast, lung, head and neck, prostate cancer, and glioblastoma. Moreover, Gal-1 expression is correlated with GBM tumor grade and malignancy [29]. Conversely, lower levels of Gal-1 expression are associated with unusually longer survival periods [30]. This correlative expression pattern is conserved in humans and mice as increased Gal-1 expression has been observed for highly invasive GBM tumors both in surgical biopsies and in mouse models. Gal-1 promotes stem cell proliferation and self-renewal in lung cancer via the Warburg effect, where cancer cells rely on glycolysis rather than on oxidative phosphorylation for glucose metabolism [31, 32].

The recent body of literature confirms that hypoxia is known to activate Gal-1 expression, and Gal-1 is under the transcriptional control of HIF-1-α [33, 34]. The hypoxic environment inherent to GBM activates HIF-1-alpha which in turn binds to gene promotors associated with tumor metabolism [35]. One of the surrogate markers of hypoxia is carbonic anhydrase IX (CA-IX), an enzyme that has been touted as a promising target for the treatment of solid cancers [36, 37]. As an acid-base regulatory protein, carbonic anhydrases catalyze the conversion of carbon dioxide and water into bicarbonate [38]. However, in GBM, CA-IX elevation is associated with a poor prognosis. It is known to reside downstream of HIF-1-α within the mitogen-activated protein (MAP) and phosphoinositide 3 kinase signaling pathway [39, 40]. The expression of CA-IX is limited in normal tissues, and its overexpression correlated with a poor prognosis has been observed in many solid malignancies, including breast, lung, ovary, head and neck, bladder, colon, cervix, renal cancers, and glioblastoma [41–44]. Besides that, CA-IX overexpression is also known to induce CSC proliferation as the mechanism of driving cancer growth [45].

The present study aims to determine the role of Gal-1 in GSCs and elucidate the potential mechanisms of Gal-1 in regulating the metabolic behaviors of GSCs. We show that Gal-1 is highly expressed both in the GBM and in patient-derived GSC. Gal-1 is under the transcriptional control of HIF-1α, and in turn, Gal-1 exerts its metabolic influence via physical association with CA-IX, the surrogate marker of hypoxia. Silencing Gal-1 reverses the Warburg effect by reducing the expression of CA-IX. Manipulation of Gal-1 in mice models showed reduced tumor growth and increased survival compared to the controls. It is the first study to demonstrate that Gal-1 plays an important role in GSC metabolism through mechanisms mediated by CA-IX. Further, targeting the Gal-1/CA-IX signaling pathway provides a new strategy for reversing the Warburg effect in GBM and inhibiting the progression of GSC-induced cancer growth.

## Materials and methods

### Ethics statement

Human GBM surgical biopsy (hGBM) specimens and normal brain tissues used in this study were obtained from the OSF Saint Francis Medical Center, Peoria, IL, and processed in accordance with the UICOMP Institutional Review Board–approved protocol (Protocol #85193).

### Cell culture and reagents

Previously established and authenticated GSCs—GSC20, GSC28, GSC262, GSC268, and GSC6-27 were obtained from Dr. Bhat’s Laboratory, MD Anderson Cancer Center, Houston, Texas. GSC33 was obtained from Dr. John Kuo’s laboratory, University of Wisconsin, Wisconsin. These GSC were grown in DMEM/F12 supplemented with B27 (Life Technologies, Carlsbad, CA), EGF (20 ng/ml) (Millipore, Sigma, St. Louis, MO), bFGF (20 ng/ml) (Millipore, Sigma), 1% penicillin/streptomycin (Life Technologies). Human astrocytes (#1800) were obtained from ScienCell Research Laboratories (Carlsbad, CA). Gal-1 and CA-IX antibodies were obtained from both Santa Cruz (Santa Cruz, CA) and Novus Biologicals (Centennial, CO). GAPDH antibodies were obtained from Santa Cruz and HIF-1α antibodies were purchased from Novus Biologicals. Antibodies, LDHA-A, LDHA-B, PDH, p38 MAPK, Caspase-9, BAD, and NF-κB were purchased from Cell Signaling Technology (Danvers, MA). Neural progenitor cells (NPC) (PT-2599) were obtained from Lonza (Basel, Switzerland) and human astrocytes (#1800) were obtained from the Sciencell laboratories, Carlsbad, CA).

### Construction of shRNA-expressing plasmid of shGal-1

For the construction of Gal-1 shRNA- expressing vector, we used the human Gal-1 sequence as the shRNA target sequence. The plasmid vector, pSilencer™ 4.1-CMV (Ambion, Austin, TX), was used to construct the shRNA-expressing vector as previously mentioned [46]. Briefly, inverted repeat sequences synthesized for Gal-1 were laterally symmetrical, making them self-complementary with a 9-bp mismatch in the loop region to help in the loop formation of the shRNA. Oligonucleotides generated were ligated to pSilencer at the *BamHI* and *HindIII* sites. The resultant clones were verified by DNA sequencing.

### Transfection and generation of stable cells using G418

Transient transfections were performed using Lipofectamine 3000 stem reagent (Invitrogen, Carlsbad, CA). Both GSC33 and GSC20 were transfected with 5 μg plasmid per plate [plasmid (μg): Lipofectamine 3000 stem (μl) = 1:3]. After 72 h of transfection, both GSC33 and GSC20 cells were selected using 800 µg/ml of G418. Around six G418-resistant single-cell clones/colonies were obtained and isolated using cloning cylinders (Millipore, Sigma) and expanded. These clones were further validated by the immunoblot approach.

### RT-PCR analysis

We isolated total RNA from both GSC33 and GSC20 stable cells expressing shGal-1 and scrambled vector. RT-PCR was conducted using the SYBR green method [47]. The primers used in the study were Gal-1 Forward 5′-CAGCTGGGTTGGTATGGAGT-3′ and Reverse 5′-GCCCTCACTCAGCCAGTAAC-3′; GAPDH Forward 5′-AATCCCATCACCA TCTTCCA-3′ and Reverse 5′-TGGACTCCACGAC GTACTCA-3’.

### RNA-seq analysis of shGal-1 and SV in GSC20

Total RNA samples isolated from the scrambled vector (SV) and shGal-1 stable cells of GSC20 were measured using Agilent 2100 Bioanalyzer (Agilent RNA 6000 Nano Kit, Santa Clara, CA). The total RNA proceeds to cDNA preparation, and the obtained single-strand DNA is then sequenced on BGISEQ-500 platform. Heat maps represent the mRNA fold changes obtained.

### MTT assay

The proliferation of GSC20 and GSC33 was measured using the 3-(4, 5-dimethylthiazol-2-yl)-2, 5-diphenyl tetrazolium-bromide (MTT) (Invitrogen; Carlsbad, CA) assay. Around 10,000 cells of both GSC20 and GSC33 were incubated with vehicle, SV, and varying concentrations of SLC-0111 (75 µM, 100 µM, 125 µM, and 150 µM), for 24 h in the 96-well plate. The plate was incubated for 2 h at 37 °C with 100 μl of MTT reagent to allow the formation of formazan crystals. We replaced the medium with dimethyl sulfoxide (0.1 ml/well) and incubated the plate for 30 minutes at room temperature. Optical density was read at 590 nm. Cell viability was calculated by normalizing absorbances against the untreated control cultures.

### Immunoblot analysis

Both untreated and treated cells in this study were lysed in RIPA lysis buffer (Boston BioProducts, Milford, MA) supplemented with proteasome and phosphatase inhibitors, and lysates were cleared by centrifugation. The Pierce BCA Protein Assay Kit (Thermo Fisher Scientific, Waltham, MA) was used to quantify total protein. Cell lysates were resolved using SDS-polyacrylamide gel electrophoresis (PAGE). Blots were incubated with respective primary antibodies, followed by incubation with horseradish peroxidase (HRP)-conjugated secondary antibodies. Immuno reactive bands were visualized using an enhanced chemiluminescent (ECL) reagent (Bio-Rad, Hercules, CA). Western blot detection reagents on Hyperfilm MP Autoradiography film (Amersham, Piscataway, NJ). Antibodies, Gal-1, CA-IX, LDHA-A, LDHA-B, PDH, p38 MAPK, Caspase-9, BAD, and NF-κB and GAPDH antibodies were diluted to 1:1000, whereas the HIF-1α antibody was used at a dilution of 1:500. GAPDH antibody was used as a loading control.

### Biolog mitoplate S1

GSC20 and GSC33 SV and shGal-1 cells were assayed in triplicate for mitochondrial activity using 96-well MitoPlate S1 plates (Biolog, Hayward, CA) that contained 31 cytoplasmic and mitochondrial metabolic substrates that produce NADH (e.g. L-malate, α- ketoglutarate, D-Isocitrate, L-glutamate, D-β-hydroxybutyrate) or FADH2 (e.g. succinate, α-glycerol-PO4). Each substrate uses different transporters to enter the mitochondria and different dehydrogenases to produce NADH or FADH_2_. The electrons travel to the distal portion of the electron transport chain where a tetrazolium redox dye (MC) acts as a terminal electron acceptor that turns purple upon reduction. Cells were treated with Saponin (MilliporeSigma, St.Louis, MO) with 40 µg/ml before an assay master mix was prepared using 2X Biolog MAS, 6x Redox Dye, and sterile water. Both control and shGal-1 stable cells were harvested and suspended in 1x Biolog MAS. The cell viability was verified using the Trypan blue dye. Around 30 µl of assay mix was added into all wells of Mitoplate S1 and was incubated at 37 °C for 1 h followed by the addition of 30 µl per well (30,000 cells/well) cell suspension to all wells. Metabolism of substrates was assessed by colorimetric change of a terminal electron acceptor tetrazolium redox dye at a wavelength of 590 nm. A metabolic score was created based on the rates of all substrates and normalized against the mtDNA content in each sample.

### Hypoxia and si-HIF-1-α treatments

GSC20 and GSC33 cells were exposed to 1 to 3 cycles of hypoxia and normoxia. Each hypoxic cycle consisted of a period of 24 hours in 1% oxygen followed by 24 h recovery under normoxia conditions. During this reoxygenation period, cells were provided with a fresh medium. In another experiment, we used HIF-1α siRNA (sc35561, Santa Cruz Biotechnology) to transfect both GSC20 and GSC33 cells. After 48 h of transfection, lysates were obtained and proceeded for further analysis using immunoblot analysis.

### GC-MS metabolic profiling

Both GSC20 and GSC33 SV-treated- and shGal-1 stable cells were collected, and the cell pellet was washed twice with ice-cold PBS. The cells were then extracted with 0.5 ml of ice-cold chloroform: methanol (2:1 v/v) and vortexed. The residues were stored at −80 °C prior to analyses.

### Immunohistochemistry of hGBM specimens and in vivo tissue sections

Representative sections of normal human brain cortex and GBM-12 specimen were DAB stained with Gal-1 and CA-IX antibodies as described previously [48]. Briefly, paraffin-embedded sections were probed with the aforementioned antibodies and then stained with DAB. Negative controls were maintained using IgG antibodies instead of the primary antibody. Using a confocal microscope, the sections were then examined according to standard protocols.

### TCGA dataset

To understand the expression and clinical outcomes of Gal-1 in GBM and normal brain tissue samples, we retrieved and analyzed the available public data using the Cancer Genome Atlas project data available on Affymetrix HT-HG-U133A array, (https://tcga-data.nci.nih.gov/tcga/) from 525 GBM cases, including gene expression data and follow-up information. We downloaded open access REMBRANDT data on 5 May 2022 using the Gliovis data portal (http://gliovis.bioinfo.cnio.es/) [49, 50]. We also obtained the gene expression and clinical data from the long-time survivors using the REMBRANDT database.

### Limiting dilution assay

For this assay, a 96-well plate pre-coated with poly d-lysine was used to plate GSC20 and GSC33 in increasing cell numbers (500, 1000, and 2000 cells/well) with 12 replicates/cell numbers. Before plating, the spheres of cells were disassociated into a single-cell suspension. Cells were treated with shGal-1. Both untreated and treated GSC20 and GSC33 were counted on the 1st, 7th, and 14^th^ day under a phase-contrast microscope, and data were analyzed using the Extreme Limited Dilution Analysis (ELDA) platform to determine stem cell frequency (http://bioinf.wehi.edu.au/software/elda/) [51].

### Cellular ATP measurement by luciferase assay

Total cellular ATP was assayed by using the ATP determination kit (Molecular Probes, Eugene, OR), following the manufacturer’s instructions. SV- treated and shGal-1 stable cells of GSC20 and GSC33 cells were lysed, and lysates were cleared by centrifugation. The Pierce BCA Protein Assay Kit (Thermo Fisher Scientific, Waltham, MA) was used to quantify total protein. Equal concentrations of protein from the SV and shGal-1-treated samples were added to the standard reaction solution and the luminescence was quantified using a GloMax® Discover Microplate Reader, Promega (Madison, WI, USA). Values were calculated based on an ATP standard curve. All ATP concentrations were determined in triplicate.

### Detection of reactive oxygen species (ROS)

ROS formation was detected using a cell-permeable fluorescent compound, 2′, 7′-dichlorofluorescein diacetate (DCFDA) (MilliporeSigma, St.Louis, MO). SV and shGal-1 stable cells of GSC20 and GSC33 cells were incubated DCFDA (20 µM, 100 µl/well) for 30 min at 37 °C in the dark. DCFDA, a non-fluorescent compound, can be oxidized by ROS into 2′,7′-dichlorofluorescin (DCF), a highly fluorescent compound whose signaling was detected by excitation and emission wavelength at 495 nm and 529 nm, respectively. This experiment was run in triplicate.

### Caspase-Glo 3/7 activity and Annexin V-FITC/PI assay

Measurements of caspase activities in stable cells of SV and shGal-1 were performed using the commercially available Caspase-Glo 3/7 Assay (Promega, Madison, WI) following the manufacturer’s instructions. The aforementioned cells were seeded in 96-well culture plates at 2500, 5000, 7500, and 10,000 cells/well. Then, 100 µl of Caspase-Glo 3/7 reagent was added to each well. Cells were mixed using a plate shaker at 300 rpm for 45 seconds and left in the dark at room temperature for 40 minutes, followed by measurement of luminescence with SpectraMax iD3 (Molecular Devices). This experiment was run in triplicate. For the radiation experiment, an IR dose of 4 Gy was given to both SV and shGal-1 cells using the RS 2000 Biological Irradiator X-ray unit (Rad Source Technologies Inc., Boca Raton, FL) as described previously [52]. Irradiated cells were further incubated for 24 h before proceeding for the Caspase-Glo 3/7 Assay. Annexin V-FITC/PI staining method was used to detect the percentage of apoptotic cells upon IR and shGal-1 treatment. Both SV and shGal-1 GSC (1×10^5^cells/ml) were treated according to the manufacturer’s instructions for ANNEX100F Kit. FITC/PI fluorescence intensity was analyzed by flow cytometry using BD FACSAria III (BD Biosciences, USA) and data was used to differentiate between viable, early apoptotic, and necrotic cells. The extent of apoptosis was quantified according to the percentage of annexin V-positive cells (*n* = 3).

### Duolink proximity ligation assay

The Duolink® In Situ Red Starter Kit (Mouse/Rabbit; DUO92101; (MilliporeSigma, St.Louis, MO) was used to perform the proximity ligation assay (PLA) in accordance with the manufacturer’s instructions and our previously published protocol [44]. Around 500–1000 GSC20 and GSC33 cells were plated onto an 8-well laminin-coated chambered slide. Both the cells were treated with Gal-1 and CA-IX antibodies, followed by the addition of the diluted PLA probes. Next, amplification was performed by incubating cells with a polymerase reaction mix. Slides were coverslipped using DAPI containing ProLong Gold mounting medium, and the micrographs were acquired using an Olympus BX61 microscope (Olympus BX61 Fluoview, Minneapolis, MN).

### BrdU (Bromodeoxyuridine) cell proliferation assay

Using a BrdU Kit (#6813, Cell Signaling Technology Danvers, MA, USA), we examined the effect of silencing shGal-1 on GSC proliferation. To synchronize the cell cycle, approximately 30,000 cells were seeded in 100 µl of medium and the cells were incubated for 24 hours at 37 °C. After switching to a 1x BrdU solution, the medium was incubated for 24 hours at 37 °C in order to induce proliferation and incorporation of BrdU. An ELISA test was performed to detect BrdU incorporation following the manufacturer’s instructions. At 450 nm, BrdU incorporation was determined using an xMark Bio-Rad microplate spectrophotometer.

### GST-galectin fusion protein preparation and pull-down

GST-tagged ORF expression clone for *LGALS1* in bacteria was purchased from Genecopoeia (EX-I0309-B04). To verify the expression of Gal-1 protein expression, we transformed this plasmid in BL21 (DE3) E. coli competent cells. Fusion protein expression was induced by adding 0.5 mM IPTG for three hours. Bacteria were lysed with 2 mg/ml lysozyme in a lysis buffer. Glutathione agarose was equilibrated in Pierce spin columns using wash buffer, followed by the addition of 200 µg of the GST fusion protein for immobilization. The spin column was placed in a gentle rocking motion on a rotating platform for 3 h incubated at 4 °C. Then, the spin column tube with the mixture was centrifuged and washed five times with a wash solution. To the bound fraction, we added 200 µg of total protein of GSC33 control cells. The GST pulldown assay was performed from the Pierce GST Protein Interaction Pull-Down Kit (Thermo Scientific) using the manufacturer’s instructions.

### Intracranial injections

Around 1 × 10^5^ GSC33 control and shGal-1 cells were stereotactically implanted into a 4-week-old athymic nude mice (nude-*Foxn1*^nu^, 4 male and 4 female mice from Envigo), lateral to the bregma in the right cerebral hemisphere by drilling into the animal’s skull with the drill, piercing only the bone, by following the previously published protocols [48, 53]. Tumor formation and the phenotype were determined by histologic analysis of hematoxylin and eosin-stained sections. Mice were euthanized when they reached moribund state or met the experimental endpoints.

### Statistical analysis

The results are represented as mean ± SD. The differences between the control and treated groups were evaluated with Student’s t-tests and ANOVA using Graph Pad 9.0. For multiple comparisons within TCGA, Student’s *t* tests were used to calculate expression differences. All *p* values were considered statistically significant with a value <0.05.

## Results

### Overexpression of Gal-1 predicts poor GBM patient survival

**Fig. 1:**
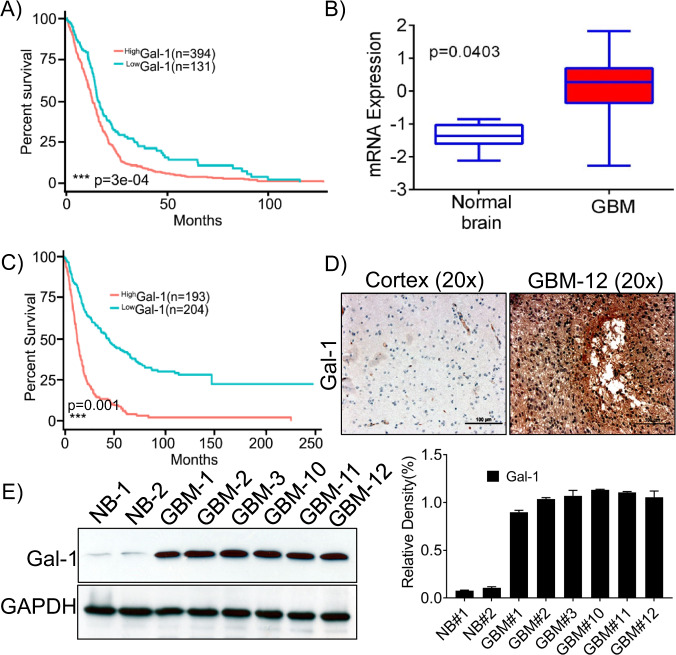
Gal-1 expression at mRNA level on GBM patient tumor tissue and normal tissue specimen is associated with poor prognosis. **A** Gal-1 expression at mRNA level was high in 394 and low in 131 tumor specimen; GBM patients with high-Gal-1 expression had poor overall survival (OS). This graph represents the data from short-term survivors (≥100 months) and was retrieved from TCGA Affymetrix HT-HG-U133A array. **B** Relative mRNA expression levels of Gal-1 in GBM and normal brain tissues were plotted in a bar graph (**C**) REMBRANDT dataset shows that Gal-1 overexpression corresponds to poor prognosis. This graph represents the data from long-term survivors (≥250 months). **D** Gal-1 was highly expressed in GBM patient specimens when compared to normal brain tissue (NB). Immunohistochemical assays were performed, and the representative photographs of Gal-1 expression were shown (×20 magnification). **E** Immunoblot analysis was performed using the lysates obtained from the normal brain (NB) and GBM patient xenografts. GAPDH was used as a control for loading. The density levels were quantified and represented as a bar graph.

Recent literature by Chou et al., Rorive et al., and others showed that Gal-1 is highly expressed in GBM and is associated with tumor progression [4, 7]. Gal-1 is considered a potential biomarker since serum Gal-1 levels are higher in GBM patients than in healthy controls and that Gal-1 overexpression is associated with poor patient survival. To identify the expression of Gal-1 in GBM, we used an unbiased approach and correlated expression with clinical outcomes data using The Cancer Genome Atlas (TCGA) database (HT_HG-U133A). We collected clinical and mRNA data from 525 GBM patients, and the aggregated patient population from the GBM dataset was dichotomized into those who expressed more Gal-1 than the sample median (*n* = 394) and those who expressed less Gal-1 than the median (*n* = 131), the high Gal-1 group portended worse survivorship. Overall survival (OS) was calculated based on gene expression. The Kaplan–Meier survival curves plotted using the lower quartile showed that patients with high-Gal-1 expression had a shorter OS when compared to those with low Gal-1 expression (Fig. 1A). Similarly, the mRNA expression data obtained from the aforementioned datasets showed that Gal-1 is highly expressed in the GBM patients when compared to the normal brain (Fig. 1B). As reported by Rorive et al., the expression levels of Gal-1 are high in high-grade astrocytic tumors in patients with short-term survival periods compared to the patients with long-term survival [7]. We further evaluated Gal-1 expression in the patients with longer OS. The mRNA and clinical data of the long-term survivors were obtained from the REMBANDT data set. Then, 397 patient samples were divided into Gal-1 high (*n* = 193) and Gal-1 low (*n* = 204) groups. We noted that patients with high-Gal-1 expression had poor overall survival (Fig. 1C). To further validate the TCGA data, we detected expression levels of Gal-1 in the surgical biopsies of GBM patients along with the tissues obtained from the autopsy of the normal brain by immunohistochemistry assay. We found Gal-1 expression to be high in the GBM specimen, while the normal brain exhibited low or no expression of Gal-1 (Fig. 1D). Next, we performed immunoblot analysis from the lysates obtained from both the normal and GBM specimen, and the immunoblots confirmed that Gal-1 is highly expressed in GBM (Fig. 1E), when compared to normal brain tissues (NB). To summarize, high expression of Gal-1 was shown to predict a worse prognosis in patients with GBM.

### Gal-1 is abundantly expressed in GSC subtypes

**Fig. 2:**
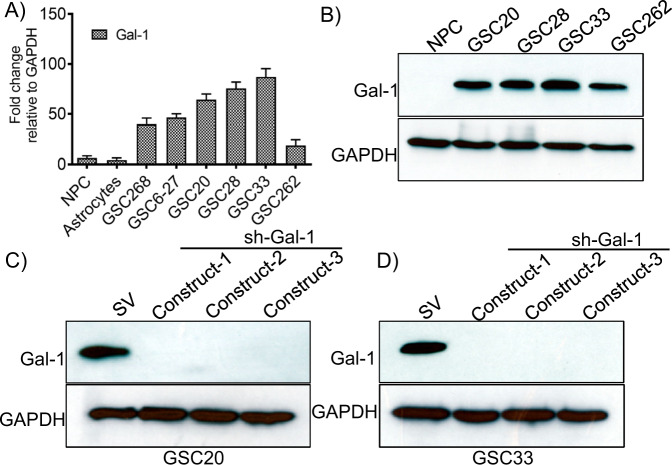
Gal-1 expression is high in PDX GBM cell lines and shRNA manipulation silences Gal-1 in GSC33 and GSC20. **A** RT-PCR analysis of Gal-1 in neuroprogenitor stem cells, astrocytes, and GSC. **B** Expression of Gal-1 in NPC and different GSC using immunoblot analysis. **C** Western blot analysis of Gal-1 expression in GSC20 using three non-overlapping shRNA constructs of Gal-1 (constructs 1, 2, and 3). All three constructs showed a significant reduction in the expression of Gal-1. **D** Gal-1 expression in GSC33 cells by shGal-1.

To verify the expression of Gal-1 in different GSC and to establish a baseline expression of Gal-1, we tested the expression of Gal-1 in short-term cultures of patient-derived xenograft (PDX) GSCs obtained from GBM patient specimens (GSC268, GSC6-27, GSC20, GSC28, GSC33, GSC262), neural progenitor cells (NPC) and astrocytes. RT-PCR analysis showed significantly higher expression of Gal-1 in the GSCs when compared to NPC and astrocytes (Fig. 2A). Next, we obtained the lysates from NPC, GSC20, GSC28, GSC33, and GSC262. Immunoblot analysis indicated that the Gal-1 expression is high in all the GSC cell lines tested (Fig. 2B) when compared to NPC. As reported previously [46] and in the methods section, we constructed shGal-1 into the pSilencerTM 4.1-CMV plasmid vector. The integration of the shGal-1 into the plasmid vector was confirmed by means of DNA sequencing. Three non-overlapping shRNA-expressing plasmid constructs of Gal-1(shGal-1) were transfected using lipofectamine 3000 and were selected using 800 µg/ml concentration of G418 after 72 h. The cells were selected for three weeks, and the G418-resistant colonies were extricated utilizing cloning cylinders and were subjected to further expansion. The western blots using the cell lysates demonstrate the complete downregulation of Gal-1 in the GSCs obtained after stable cell expansion (Fig. 2C, D).

### The shGal-1 treatment causes metabolic perturbations in GSC

To identify the global role of Gal-1 in GBM and to identify differentially expressed genes upon silencing of Gal-1, we performed RNA sequencing analysis in GSC20 cell line. RNA was isolated from the biological duplicates of scrambled vector (SV)-treated GSC20 cells and GSC20 cells stably expressing shGal-1. Hierarchical clustering between the differentially expressed genes (DEGs) separated SV and shGal-1-treated groups. Further DEGs were identified using a Wilcoxon rank-sum test with a minimum of 1.5-fold change and *p* < 0.05. The hierarchical clustering of the expressed genes was done using the Euclidean distance matrix. We identified 684 differentially expressed down regulated genes and 693 up-regulated genes comparing SV-treated to shGal-1 cells (Supplementary Figure 1A). We identified that genes involved in promoting glycolysis are significantly down-regulated, while the genes that promote oxidative phosphorylation were observed to be increased. In addition, Gal-1 silencing showed significant enrichment in various gene ontology domains and their biological process associated with negative regulation of cancers, cellular processes, and metabolism. Gene ontology (GO) enrichment analysis was performed using Cytoscape (http://cytoscape.org/) tool in order to identify biologic pathways, networks, and functional categories of differentially expressed genes. As shown, silencing Gal-1 altered cell signaling, signal transduction, tumor metabolism, and other cellular functions. In the context of GSC, these significantly altered functions may provide us with additional insight regarding Gal-1’s role (Supplementary Figure 1B).

**Fig. 3:**
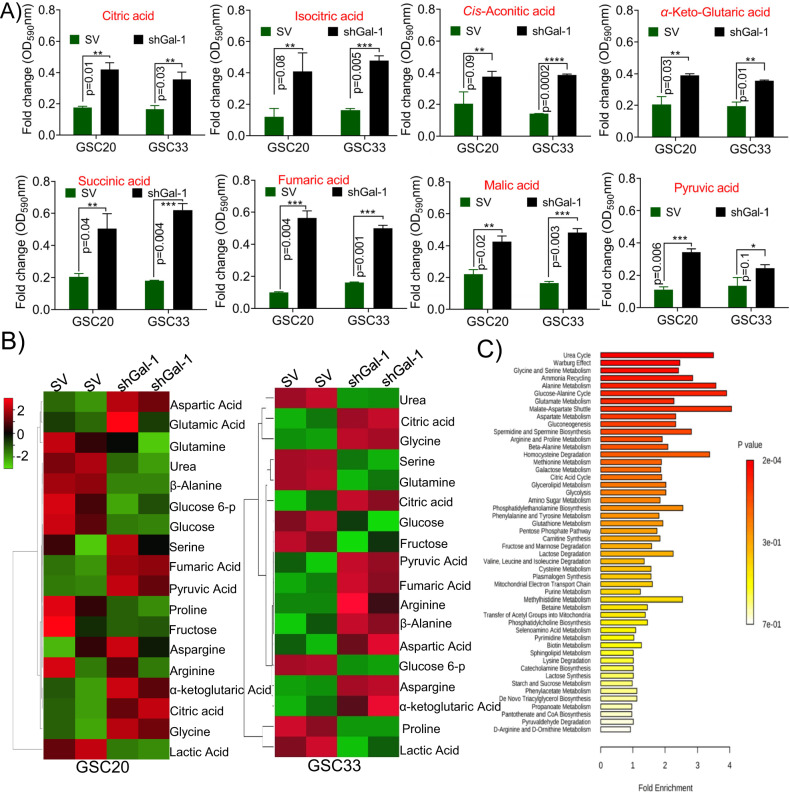
Metabolite analysis upon Gal-1 manipulation in GSC. **A** Biolog’s MitoPlates S1 (14105, Biolog) study was performed as per the manufacturer’s instruction. The data obtained from SV and shGal-1 treatment from both GSC20 and GSC33 were presented as a bar graph. **B** Evaluation of the metabolic profile in GSC20 and GSC33 using GC/MS analysis. The fold changes of different endogenous metabolites in SV and shGal-1 in GSC20 and GSC33 cells were plotted in a heatmap. **C** KEGG pathway fold enrichment analysis of all different endogenous metabolites.

To examine changes in global energy-producing metabolism in GSC with silencing of Gal-1, we performed a Mitoplate assay. Using colorimetric assay, the rate of electrons flowing into and through the electron transport chain from metabolic substrates is measured to assess mitochondrial function. For this experiment, 30,000 cells of GSC33, GSC20, SV- and shGal-1-treated cells were permeabilized with 40 µg/ml Saponin and calorimetrically evaluated. We observed that the shGal-1 stable cells showed increased expression in the levels of citric acid, isocitric acid, *cis-*aconitic acid, α-ketoglutaric acid, succinic acid, fumaric acid, and malic acid, the markers implicated in the tricaboxylic acid (TCA) cycle when compared to the SV-treated cells (Fig. 3A). Next, we performed mass spectrometry (MS) coupled with gas chromatography (GC) to further elucidate the metabolic pathways regulated by Gal-1 in GSC and provide insights into the metabolite analysis in both SV- and shGal-1 treatments. We observed specific sets of metabolites were altered considerably in shGal-1 compared to SV-treated cells. The heatmap of the top 18 significantly changed metabolites is shown in Fig. 3B. The levels of metabolites citric acid, fumaric acid, aspartic acid, α-ketoglutaric acid were significantly higher in shGal-1 cells when compared to SV-treated GSC20 and GSC33, which were consistent with the Mitoplate data. Furthermore, a significant reduction in lactic acid, glucose, fructose, and proline levels was observed in the shGal-1 treatments. Additionally, enrichment analysis further demonstrated that altered metabolites were involved in the Warburg effect, glycolysis, gluconeogenesis, urea cycle, glutamate metabolism, citric acid cycle, aspartate metabolism, purine, and pyrimidine metabolism (Fig. 3C), which further confirmed the important role of targeting Gal-1 in understanding GSC metabolism. These results infer the modulation of Gal-1 invokes a suite of metabolic processes that facilitate the reversal of the Warburg effect in GSC.

### Targeting Gal-1 attenuates GSC self-renewal, proliferation, and reverses the Warburg effect

**Fig. 4:**
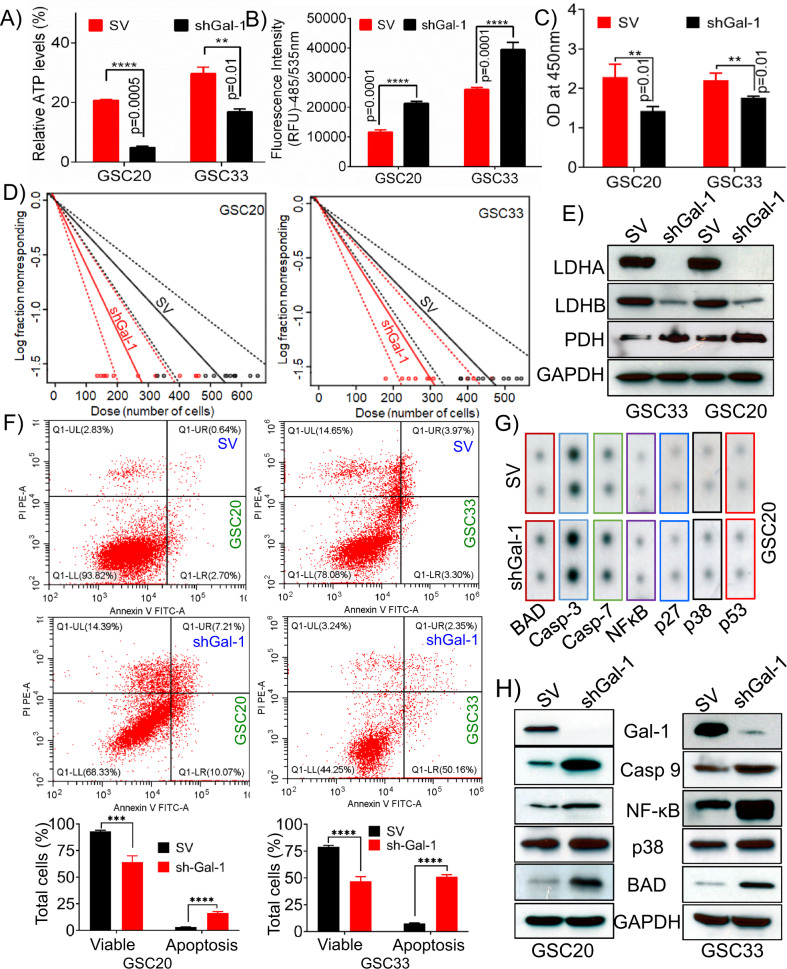
Silencing Gal-1 reverses the Warburg effect and induces apoptosis. **A** Cellular ATP in SV and shGal-1 stable cells in GSC20 and GSC33 cell lines. **B** DCFDA staining was used to determine ROS levels in SV and shGal-1 treatments in both GSC20 and GSC33. **C** BrdU assay to verify the cell viability in both SV and shGal-1 cells. **D** Limiting dilution assays to study the GSC proliferation in SV and shGal-1-treated GSC20 and GSC33 cells. Each result is representative of three independent experiments. **E** Immunoblot analysis of different glycolytic enzymes in SV and shGal-1-stable cells of GSC33 and GSC20. **F** GSC20 and GSC33 were analyzed for Annexin V/APC and PI-induced apoptosis following manipulation of shGal-1. Dot plots (Red dots) and bar graphs represent the percentage of healthy, necrotic, early apoptotic, and late apoptotic cells in GSC20 and GSC33 cells. These data were compared with those in the control group. **G** Human apoptosis signaling pathway array (Ray Biotech AAH-APOSIG), to verify the induction of apoptosis in the shGal-1-stable cells when compared to the SV in GSC20. **H** Immunoblot analysis of various apoptosis markers in shGal-1-stable cells.

**Fig. 5:**
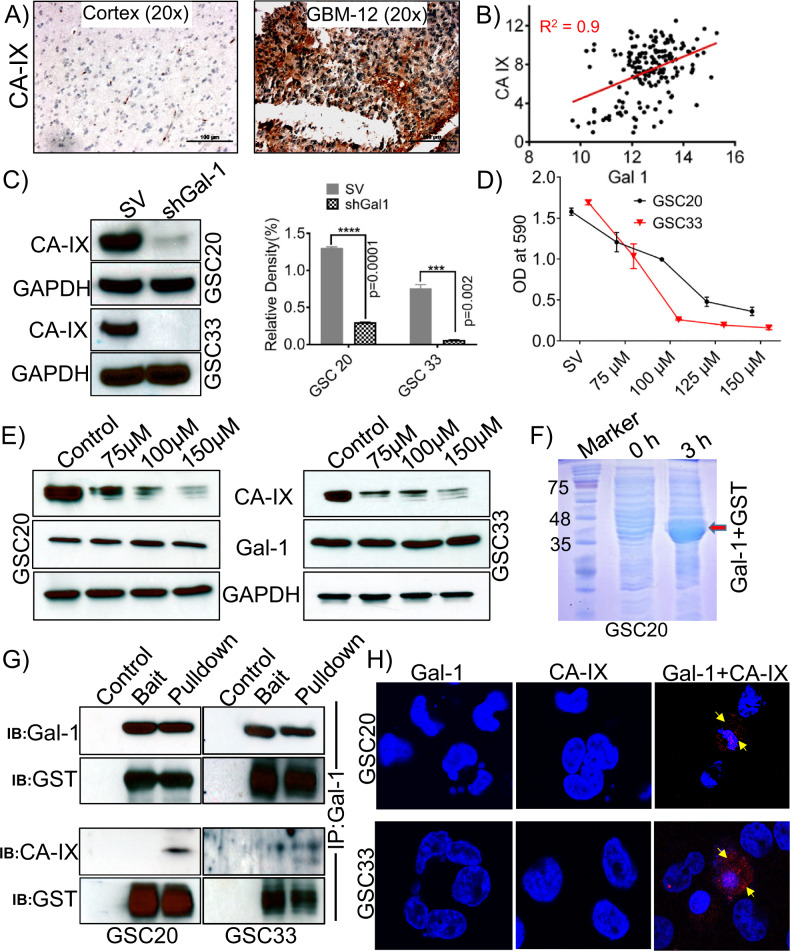
Carbonic anhydrase (CA-IX) is highly expressed in GBM; Gal-1 regulates the expression of CA-IX. **A** Immunohistochemical staining images of CA-IX expression in control brain and GBM specimen (Scale bar-100 μm). **B** The mRNA obtained for Gal-1 and CA-IX from TCGA datasets showed a positive correlation. **C** Western blot analysis of CA-IX in shGal-1 stable and SV treated GSC20 and GSC33 cells. The density levels were quantified and represented as a bar graph. **D** MTT assay. **E** Expression of CA-IX and Gal-1 in untreated controls and samples treated with different concentrations of CA-IX inhibitor, SLC-0111, in GSC20 and GSC33 cells. **F** Coomassie Blue-stained SDS-PAGE gel showing the expression levels of GST-Gal-1 protein after induction with 0.5 mM IPTG for 3 h. **G** Co-immunoprecipitation (GST pulldown) assay was performed with Gal-1 and CA-IX antibodies, using GST-tagged Gal-1 fusion purified protein lysate as prey and GSC20/GSC33 control total protein as bait. Eluted proteins were resolved by electrophoresis and subjected to immunoblot analysis. GST is shown as a loading control. **H** PLA of Gal-1 and CA-IX were performed in GSC20 and GSC33 cells. The representative micrograph of Gal-1 + CA-IX shows the positive PLA. Red spots confirmed the association. Single antibody stain with Gal-1 and CA-IX showed negative results. DAPI was used to stain the nuclei.

To delineate the function and metabolic role of Gal-1 in GSC metabolism, we performed ATP production assay on GSC20 and GSC33 cells. The shGal-1-stable cells demonstrated attenuated respiration when compared to the SV control (Fig. 4A). Previous reports implicate the role of oxidative stress serves in the induction of cellular apoptosis [54, 55]. Next, we performed intracellular ROS production by using DCFDA staining, and the quantification was done by calculating the excitation and emission at 485/535 nm optical density. We observed increased levels of ROS production in shGal-1 stable cells compared to SV-treated cells (Fig. 4B). As we observed decreased levels of ATP and increased ROS production, we next checked for cell growth and proliferation using BrdU assay. We confirmed that there was a reduction in cell viability and proliferation in the shGal-1-treated cells when compared to the SV treatments (Fig. 4C). Next, we performed the limiting dilution assay on SV- treated and shGal-1 stable cells of GSC20 and GSC33 and showed reduced self-renewal in the shGal-1 stable cells when compared to SV-treated cells (Fig. 4D). Immunoblot analysis showed increased expression levels of PDH with a concomitant reduction in the expression levels of Lactate Dehydrogenase A (LDHA), and Lactate Dehydrogenase B (LDHB), indicating the reversal of the Warburg Effect (Fig. 4E). Considering that the manipulation of Gal-1 decreased the levels of ATP and cell viability, we performed flow cytometric analysis using Annexin V-FITC/PI to determine if there were significant levels of apoptosis in GSC. As compared to SV, shGal-1 treatments resulted in an increase in Annexin V-positive apoptotic cells in GSC20 and GSC33. In GSC20 shGal-1-treated cells, the proportion of apoptotic cells increased from 3.34% to 17.28%, whereas in GSC33 shGal-1-treated cells, the percentage increased from 7.27% to 52.51%. According to these findings, manipulation of Gal-1 leads to increased apoptosis in GSC20 and GSC33 (Fig. 5F). Induction of apoptosis and apoptotic response profiles were further assessed using a human apoptosis signaling pathway array (Ray Biotech AAH-APOSIG). We recorded increased expression levels of apoptotic markers such as BAD, Caspase-3, Caspase-7, NF-kB, p27, p38 MAPK and p53 in the shGal-1 stable- cells when compared to the SV treatments (Fig. 4G). We next performed immunoblot analysis to verify the expression of independent markers, and the results (Fig. 4H) corroborated with the data from Fig. 4G. We next examined whether the silencing of Gal-1 leads to apoptosis when challenged with an apoptotic stimulus. As a stimulus, we used irradiation for the induction of cell death [56]. We irradiated both the GSC20 and GSC30 SV and shGal-1 cells with a dose of 4 Gy. To further confirm the induction of apoptosis, we performed quantitative Caspase activity using the luminescence detection method. We recorded a linear increase in the caspase-3/7 activity with an increased number of cells in both shGal-1 and shGal-1 + 4 Gy samples compared to SV treated GSC20 and GSC33 (Supplementary Figure 2). Collectively the results presented confirm that Gal-1 promotes glycolysis, and silencing Gal-1 reverses the Warburg effect and can compromise the GSC phenotype and replication.

### Carbonic anhydrase IX (CA-IX) is a target of Gal-1 signaling, and silencing HIF-1-α reduces Gal-1-CA-IX association

Previously published literature confirms that high CA-IX expression is an independent prognostic marker for poor survival among patients with glioblastoma [42]. RNA sequencing analysis showed reduced levels of CA-IX in the shGal-1 stable cells when compared to the SV treatments (Supplementary Figure 1A). Since CA-IX plays an essential role in the tumor metabolism and promotion of glycolysis, we next sought to understand if Gal-1 regulates CA-IX expression. We first verified the expression of CA-IX in our cohort of GBM specimens and observed that CA-IX is significantly expressed in GBM specimens, and no expression is recorded in the normal human brain specimen (cortex) (Fig. 5A). Next, we analyzed the expression correlation between CA-IX and Gal-1 in TCGA dataset, and the data indicated that CA-IX presents a statistically significant positive correlation with Gal-1(Fig. 5B). The immunoblot analysis confirmed a significant reduction in the levels of CA-IX in shGal-1 stable cells when compared to the SV-treated cells, implicating that Gal-1 may influence the expression of CA-IX in GSC (Fig. 5C). To determine if CA-IX also regulates Gal-1 expression, we used ureido-substituted benzenesulfonamide (SLC-0111), a novel clinical carbonic anhydrase inhibitor that is in phase I clinical trials (NCT02215850). The targeting of CA-IX activity with SLC-0111, alone or in combination with chemotherapy or immune checkpoint blockade, has been demonstrated in preclinical studies in a variety of solid tumor models to have antitumor activity [57]. To determine the pharmacological effects of SLC-0111, both GSC20 and GSC33 cells were treated at different concentrations of SLC-0111 (75, 100, 125, and 150 µM). In order to measure the viability of the cells upon various treatments, we performed an MTT assay. Results showed a significant dose-dependent decrease in cell viability following the treatment with SLC-0111 compared with the untreated controls (Fig. 5D). Interestingly, the highest level of inhibition was observed following treatment with 150 µM, SLC-0111. Subsequent immunoblot analysis was performed to determine whether CA-IX expression levels were inhibited in SLC-0111 treatments. According to the immunoblot results, the CA-IX expression was significantly reduced in a dose-dependent manner. Gal-1, on the other hand, displayed no change in the SLC-0111- treated GSC20 and GSC33 (Fig. 5E). In light of the expressional correlation between Gal-1 and CA-IX in Fig. 5B, we next investigated whether Gal-1 physically associates with CA-IX in GSC20 and GSC33. In order to confirm the interaction, we performed co-immunoprecipitation experiments. We first transformed GST-tagged Gal-1 in E. coli and then induced it with IPTG for 3 h. As shown in Fig. 5F, the Coomassie blue-stained acrylamide gel confirms the induction of Gal-1 protein when compared with the un-induced control. We then performed a GST pull-down assay to determine whether Gal-1 binds to CA-IX. It was found that CA-IX physically interacts with Gal-1 in both GSC20 and GSC30 cells. GST protein expression was used as a loading control (Fig. 5G). In order to test the in situ interaction between CA-IX and Gal-1, we used PLA. Using this assay, we determined if there is a direct interaction between CA-IX and Gal-1, as oligonucleotides conjugated to the CA-IX and Gal-1 antibodies produce circular DNA chains when the antibodies are in close proximity to one another. The circular DNA resulting from this reaction is amplified and detected. Using antibodies against CA-IX and Gal-1, we observed significant PLA signals (red spots) in both GSC20 and GSC33 cells. Interestingly, CA-IX and Gal-1 antibodies when individually probed did not show a PLA interaction signal (Fig. 5H). These results collectively indicate that CA-IX positively interacts and associates with Gal-1 in GSC.

### Gal-1 manipulation impairs the tumorigenic capability of GSC in mice models

**Fig. 6:**
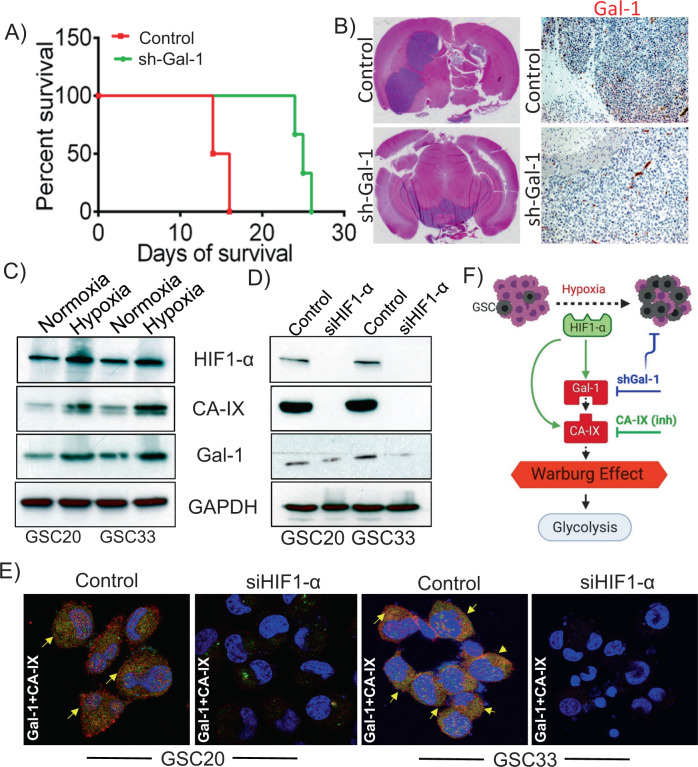
Silencing Gal-1 suppresses the growth of GSC33 generated tumor in athymic nude mice. Approximately 50,000 SV-treated and GSC33 shGal-1 cells were intracranially implanted into the brain of 4-week-old male and female athymic nude mice using stereotaxic apparatus (*n* = 8). **A** Kaplan–Meier curves plotted to show a significant increase in percent survival for all shGal-1-treated mice (*n* = 8) (*p* < 0.05) compared to untreated tumor-bearing mice (*n* = 8). **B** Hematoxylin and Eosin staining confirmed the survival data. Immunohistochemistry analysis performed on untreated and shGal-1-treated mice showed significant reduction in the expression of Gal-1 (Bar = 100 µm). **C** Immunoblot analysis shows the expression profiles of HIF-1α, Gal-1, and CA-IX in normoxia and hypoxia conditions. **D** GSC20 and GSC33 cells were transfected with siRNA against HIF-1α and the indicated proteins were analyzed by immunoblot analysis. **E** The siHIF-1α transfected GSC20 and GSC33 cells were labeled with Gal-1 and CA-IX antibodies. Immunocytochemistry shows increased colocalization of Gal-1 and CA-IX in both the controls whereas the siHIF-1α cells showed a reduced expression (Red = Gal-1; Green = CA-IX; Blue = DAPI; colocalization = Yellow). **E** Schematic representation of the possible regulation of HIF-1α-Gal-1-CA-IX axis in promoting Warburg effect in GSC.

Using athymic nude mice, we performed intracranial xenografts of control or shGal-1-expressing GSC33 in order to investigate the effects of Gal-1 manipulation on brain tumor formation. Mice receiving control GSC33 developed brain tumors 18 days after GSC33 implantation, and they met the experimental endpoint in terms of major weight loss and neurological symptoms. Interestingly, mice implanted with shGal-1 GSCs had a reduced tumor burden for over 4 weeks. Kaplan–Meier survival plots showed that the mice implanted with shGal-1 expressing cells lived longer by more than three weeks when compared to animals injected with GSC33 (Fig. 6A). As shown in Fig. 6B, immunohistochemical analysis using Gal-1 antibody detected increased Gal-1 expression in control mice brain section compared to shGal-1 mice brain sections, implicating that Gal-1 could be a regulator of GSC tumorigenesis. The reduction in the tumor was shown using Hematoxylin and Eosin staining (Fig. 6B). The results obtained in all the animals used for the study were summarized in Supplementary Table 1. The Gal-1 expression has been shown to be influenced by hypoxia, and is a direct target of the hypoxia-inducible factor (HIF-1α protein) [58, 59]. Numerous reports confirm that hypoxia promotes the Warburg effect [60–62]. As HIF-1α is a critical marker to verify the induction of hypoxia, we first verified the expression of HIF-1α in both normoxic and hypoxic conditions. In GSC20 and GSC33 cells, we observed an increase in HIF-1α, CA-IX, and Gal-1 expression levels in hypoxic conditions (Fig. 6C). To examine whether manipulating HIF-1α leads to a change in the expression of Gal-1 and CA-IX, we transfected siRNA specific to HIF-1α into both GSC20 and GSC33 cells. Transfected cells with siHIF-1α exhibited a marked reduction in HIF-1α protein levels. Furthermore, the silencing of HIF-1α reduced Gal-1 and CA-IX proteins (Fig. 6D). Next, we performed colocalization studies in the siHIF-1α transfected cells and found that there was a significant reduction in the association between Gal-1 and CA-IX in comparison to the control cells (Fig. 6E). The data suggest that the expression of Gal-1 and CA-IX are both modulated by the expression of HIF-1α. In summary, the schematic representation shows that hypoxia induces Gal-1, while Gal-1 promotes the Warburg effect by mediating its metabolic association with CA-IX and regulation of its expression in GSC (Fig. 6F).

## Discussion

A growing body of research substantiates metabolic reprogramming in GBMs as a key factor secondary to hypoxic induction factors [63, 64]. The purpose of the current study is to examine Gal-1 as a novel target for modulating GSC metabolism. This study showed that Gal-1 is avidly expressed in patients with GBM, subgroups of long-term survivors, established GBM cell lines, and patient-derived GBM tissue specimens. We demonstrated that Gal-1 functions as a metabolic modulator and an oncogene driving GSC tumor growth. Our study reports a novel interaction between Gal-1 and CA-IX for the first time. We also demonstrate that modifying the expression of CA-IX by targeting Gal-1 reverses the Warburg effect in both in vitro and in vivo models. Galectins have received considerable attention recently in cancer research and have been reported as oncogenes [29, 65, 66] but their mechanism of action is still unknown.

A study conducted on 41 high-grade astrocytoma patients found that the overexpression of Gal-1 was associated with poor overall survival [4]. Although Gal-1 overexpression has been observed in these patients, no other studies have explored its biological function and oncogenic role. In our study, we show definitive upregulation of Gal-1 in GBM specimens derived from patients of our cohort in comparison with that of the TCGA cohort dataset. Interestingly and importantly, patients with high-Gal-1 expression in both the TCGA and REMBRANDT cohorts showed decreased survival compared to the patients with low expression. Similarly, Woensel et al. reported similar findings with GBM patients’ TCGA database data. Patients with lower Gal-1 had a more favorable Th1/Treg or CTL/Treg balance, resulting in a better prognosis [67]. Based on these data, Gal-1 could be targeted to stop GSC progression. Gal-1 levels were elevated both at the transcriptional and translational levels by qRT-PCR and immunoblotting results. Our PCR data was in the line of findings with the TCGA analysis that the expression levels of Gal-1 were negligible in both NPC and astrocytes, in which Gal-1 levels were negligible.

GSCs metabolize glucose into lactate even in the presence of oxygen by using aerobic glycolysis, also known as the “Warburg effect” [68–72]. It is unclear whether targeting Gal-1 directly affects cancer metabolism, despite previous studies emphasizing its importance in cancer. Through this study, we demonstrated that manipulating Gal-1 negatively impacted glycolysis and increased the expression of genes that promote oxidative phosphorylation. Analysis of the mass spectrometry results and the mitoplate experiments between untreated Gal-1 and silenced Gal-1 cells indicates a reduction in the expression of amino acids consistent with the Warburg effect. Interestingly, some metabolites that are part of the TCA cycle and urea cycle intermediates, nucleotides were found to be up-regulated upon Gal-1 silencing [73, 74]. We observed increased levels of citric acid and alpha-ketoglutaric acid in Gal-1 silenced cells, suggesting a reversal of the Warburg effect as the decreased citrate production by the TCA is linked to tumor aggressiveness [75]. Reduced lactate levels further promote the rapid transfer of glycolytic metabolites to oxidative phosphorylation. We observe similar findings to numerous studies that show that decreased levels of genes associated with TCA cycle promote the Warburg effect and tumor growth in GBM [76, 77]. The results are in agreement with previous studies indicating that cytosolic citrate directly inhibits glycolysis to promote gluconeogenesis. The findings from this study provide a new understanding of the role of Gal-1 in GSC metabolism. Further experiments conducted confirmed the anti-Warburg effect in GSCs upon Gal-1 manipulation. In the present study, we have shown for the first time that silencing Gal-1 caused a reduction in the levels of ATP and the induction of apoptosis, as evident from the increased caspase activity. The bias toward Kreb’s cycle intermediates is a phenomenon in that levels of ATP paradoxically decline, but induction of apoptosis is seen with the known rise in caspases. Our study found that silencing Gal-1 attenuated the proliferation of GSCs and inhibited their neurosphere formation. As a result of this simultaneous increase in OXPHOS activity, ROS accumulated in the GSC, leading to increased apoptosis. To the best of our knowledge, this is the first study to demonstrate the effect of targeting Gal-1 in GSC.

The hypoxic core in GBM acts as a proliferative trigger with cascades dependent upon HIF-1 activation [78–80]. Given the sequence of events, we established that CA-IX molecule acts as a downstream target of Gal-1 that suppresses its function. A previous report by Proescholdt et al. concluded that high expression of CA-IX was associated with a poor overall survival rate among GBM patients [42]. CA-IX is widely expressed in patients with GBM and almost nonexistent in normal brain tissue suggesting, a viable target for GBM treatment. In addition, Cetin et al. found that CA-IX was also associated with a poorer survival outcome in both univariate and multivariate analysis in a cohort of 66 patients [81]. The authors confirmed that CA-IX expression was detected in tumor tissue but not in normal brain tissue. As with our data, these results support that our cohort highly expresses CA-IX. Our RNA sequencing analysis discovered that silencing Gal-1 significantly reduced CA-IX expression. An expression correlation plot using TCGA mRNA data for CA-IX and the Gal-1 gene showed a linear increase in CA-IX expression when the Gal-1 gene was expressed. Next, we tested SLC-0111, an ureido-substituted benzenesulfonamide CA-IX inhibitor, and observed no effect on Gal-1 expression, further supporting the hypothesis that Gal-1 is an upstream regulator of CA-IX. In additional pulldown experiments, it was confirmed that CA-IX is indeed the bona fide target of Gal-1. To confirm our findings, we used an intracranial murine model of GBM, where we demonstrated reduced gal-1 expression is associated with a significant survival benefit. The median survival rate shifted upwards by over 30%, with effects upon long-term survival. Finally, we conclude that both Gal-1 and CA-IX are under the transcriptional control of HIF-α.

In summary, our results demonstrate that metabolic reprogramming can be controlled by Gal-1 expression in GSCs, thereby establishing Gal-1 as a potential therapeutic target. In addition, we showed that targeting Gal-1 in vitro and in vivo had anticancer potential. In this report, we demonstrate for the first time that Gal-1 regulates CA-IX expression in GSC and that the Gal-1/CA-IX complex is responsible for metabolic reprogramming of GSC. Our findings indicate a novel interplay between Gal-1 and CA-IX, highlighting the relevance of therapeutically targeting the Gal-1/CA-IX complex in GSC. Furthermore, our results confirm that Gal-1 and CA-IX play an essential role in the modulation of the energy metabolism of GSCs.

## Supplementary information

Supplementary Information

Supplementary Figure 1

Supplementary Figure 2

Supplementary Table 1

Reproducibility Checklist

Raw data

## Data Availability

All data and materials used in the study are available in the manuscript.
