# Supplementary material for: Galectin-1 activates carbonic anhydrase IX and modulates glioma metabolism
Source: Cell Death Dis. 2022 Jun 30;13(6):574. doi: 10.1038/s41419-022-05024-z (PMC9247167; doi:10.1038/s41419-022-05024-z)
Supplement: Supplementary file 1 — Supplementary Information [file 41419_2022_5024_MOESM1_ESM.docx]

**Supplementary Information**

**Supplementary Figure-1:** RNA sequencing analysis (A) Hierarchical clustering RNA sequencing (RNA-seq) data from the scrambled vector (SV) and shGal-1-treated GSC20 cells. The gradient legend at the top right of the graph represents the FPKM (fragments per kilobase of exon model per million reads mapped). Each column represents a sample, and each row represents a gene; different colors represent different expression levels—red for high expression and blue for low expression (B) KEGG pathway enrichment analysis of the differentially expressed genes.

**Supplementary Figure-2:** Caspase3/7 activity assay: For this experiment, around 2500, 5000, 7500 and 10000 cells of SV, SV+4Gy (IR), shGal-1, shGal-1+4Gy (IR) from both the GSC20 and GSC33 were seeded into a 96 well plate. The Caspase-Glo 3/7 Reagent was added directly to cells to a final volume of 200μl per well. Then, 100 µl of Caspase-Glo3/7 reagent was added to each sample. Cells were mixed using a plate shaker at 300 rpm for 45 seconds and left in the dark at room temperature for 40 minutes, followed by measurement of luminescence with SpectraMax iD3 (Molecular Devices). Each point represents the average of 5 wells. The blank control value has been subtracted from each point.

**Supplementary Table 1:** Table representing the results obtained for the mice studies.
