# Supplementary figures and images for: Galectin-1 activates carbonic anhydrase IX and modulates glioma metabolism
Source: Cell Death Dis. 2022 Jun 30;13(6):574. doi: 10.1038/s41419-022-05024-z (PMC9247167; doi:10.1038/s41419-022-05024-z)

# Supplementary Figure-1

A)

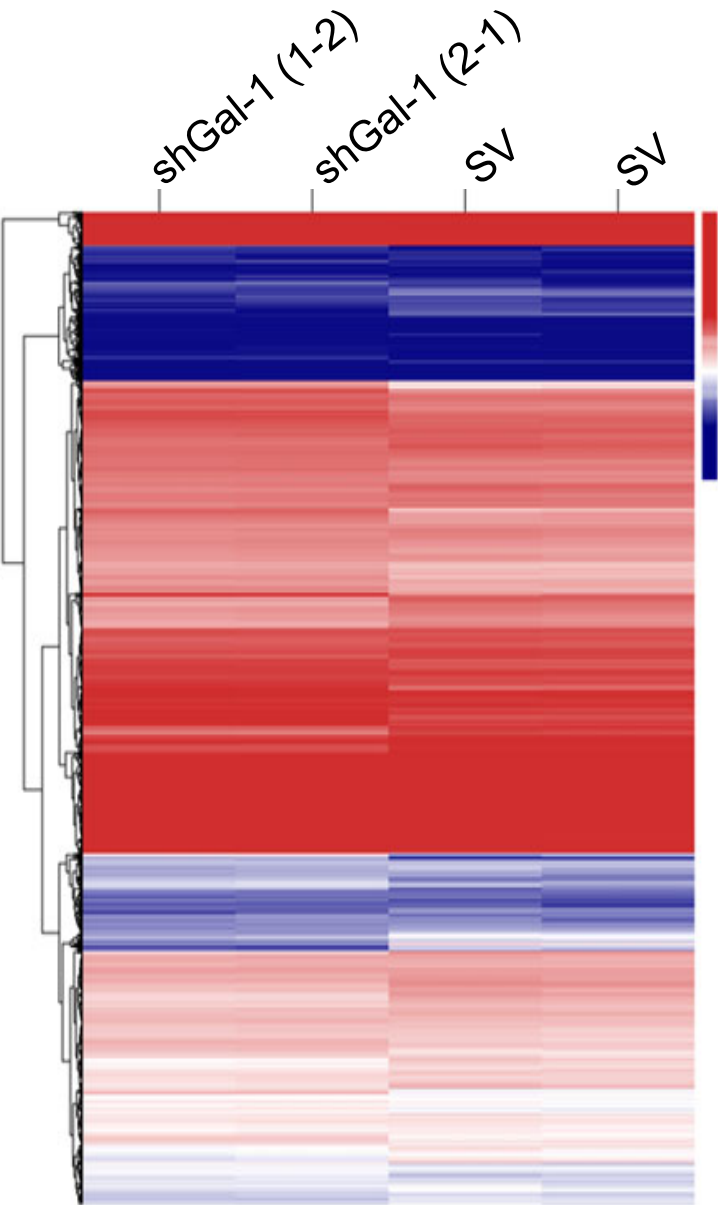

B)

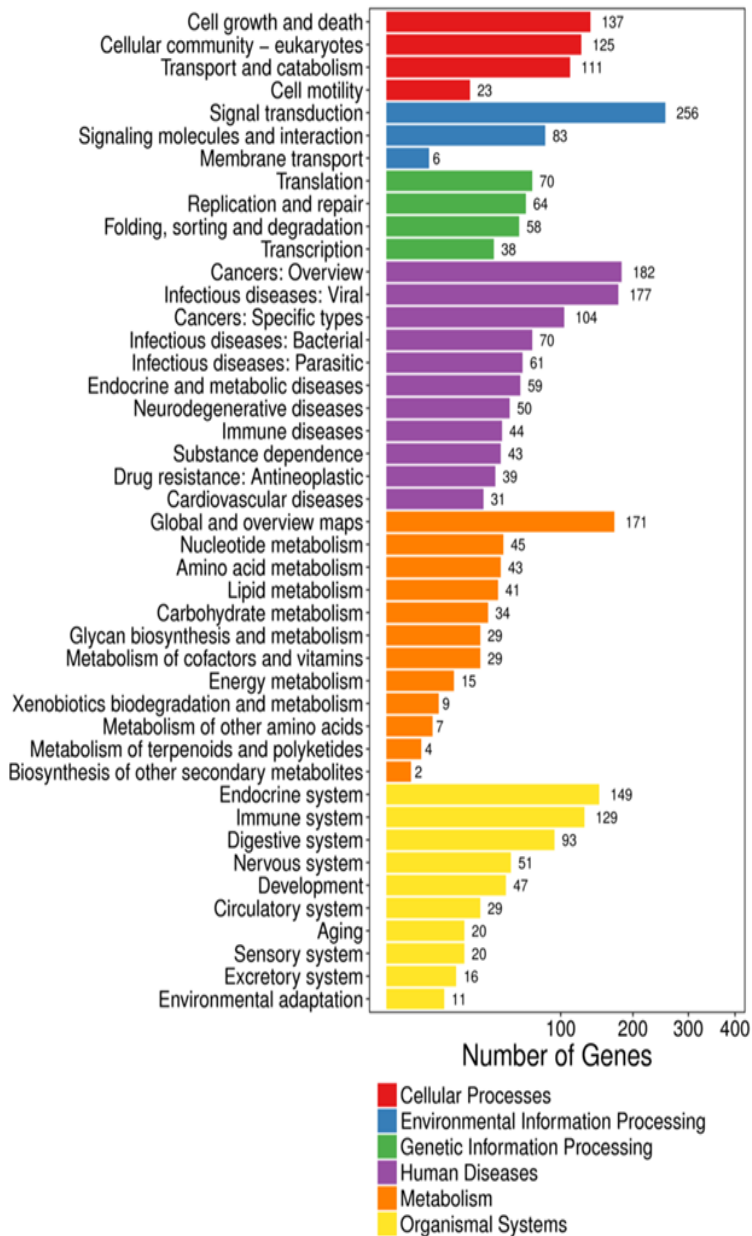

Supplement: Supplementary file 2 — Supplementary Figure 1 [file 41419_2022_5024_MOESM2_ESM.pdf]

Supplementary Figure-2

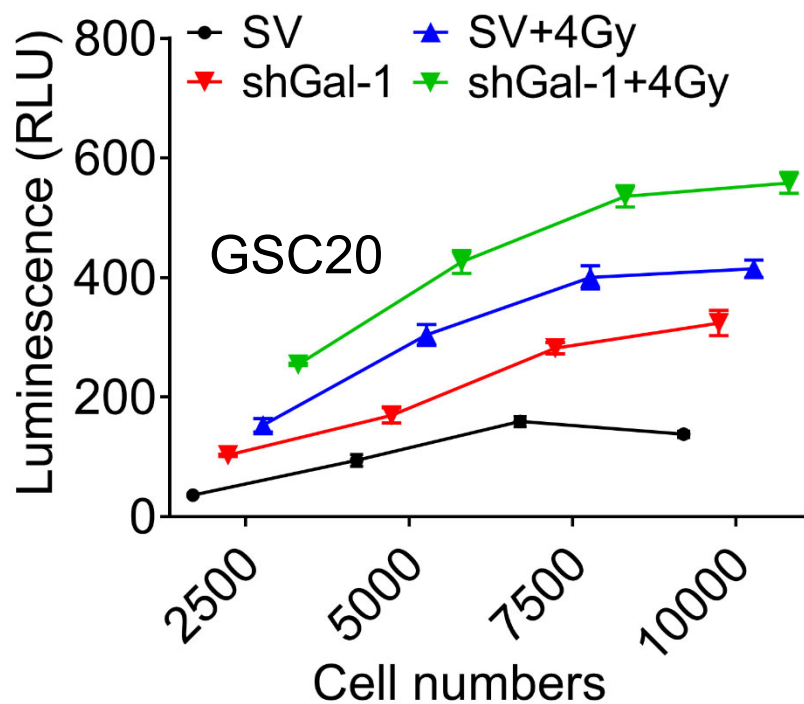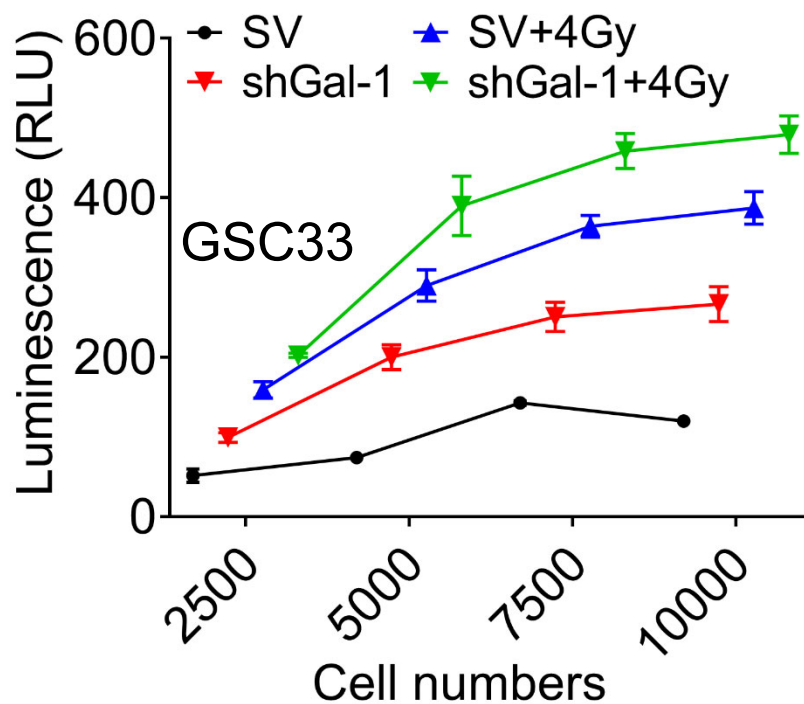

Supplement: Supplementary file 3 — Supplementary Figure 2 [file 41419_2022_5024_MOESM3_ESM.pdf]
