# Supplementary material for: Galectin-1 activates carbonic anhydrase IX and modulates glioma metabolism
Source: Cell Death Dis. 2022 Jun 30;13(6):574. doi: 10.1038/s41419-022-05024-z (PMC9247167; doi:10.1038/s41419-022-05024-z)
Supplement: Supplementary file 4 — Supplementary Table 1 [file 41419_2022_5024_MOESM4_ESM.docx]

Animal data:

|  | No. of Animals | Survival days | Tumor |
| --- | --- | --- | --- |
| SV | Male | 12 | Positive |
|  | Male | 13 | Positive |
|  | Male | 16 | Positive |
|  | Male | 18 | Positive |
|  | Female | 12 | Positive |
|  | Female | 17 | Positive |
|  | Female | 18 | Positive |
|  | Female | 17 | Positive |
|  |  |  |  |
| shGal-1 | Male | 27 | Negative |
|  | Male | 28 | Negative |
|  | Male | 30 | Negative |
|  | Male | 28 | Negative |
|  | Female | 28 | Negative |
|  | Female | 27 | Negative |
|  | Female | 30 | Negative |
|  | Female | Died during Surgery |  |
