# Supplementary material for: Galectin-1 activates carbonic anhydrase IX and modulates glioma metabolism
Source: Cell Death Dis. 2022 Jun 30;13(6):574. doi: 10.1038/s41419-022-05024-z (PMC9247167; doi:10.1038/s41419-022-05024-z)
Supplement: Supplementary file 6 — Raw data [file 41419_2022_5024_MOESM6_ESM.pptx]

## Slide 1
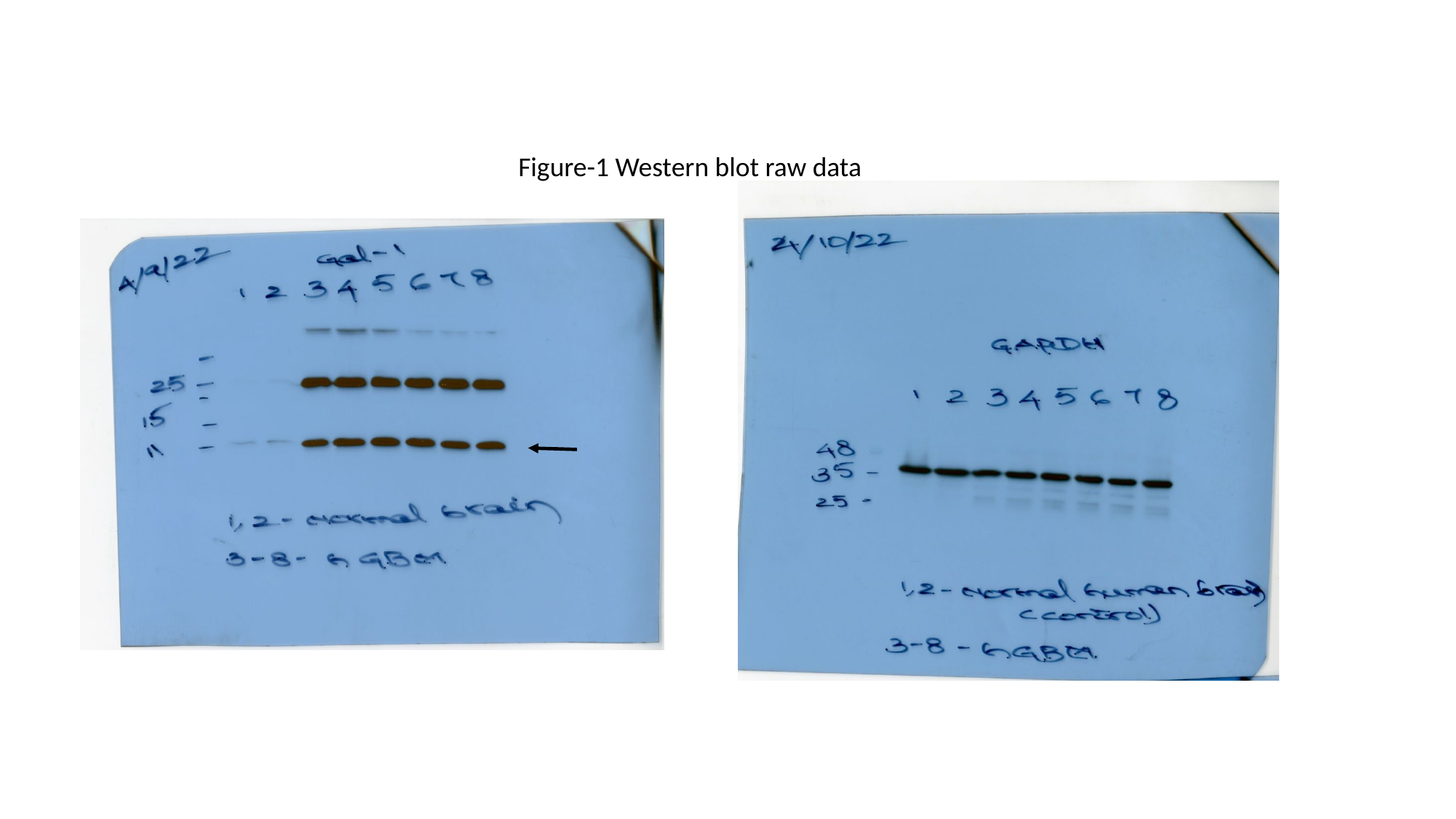

Figure-1 Western blot raw data

## Slide 2
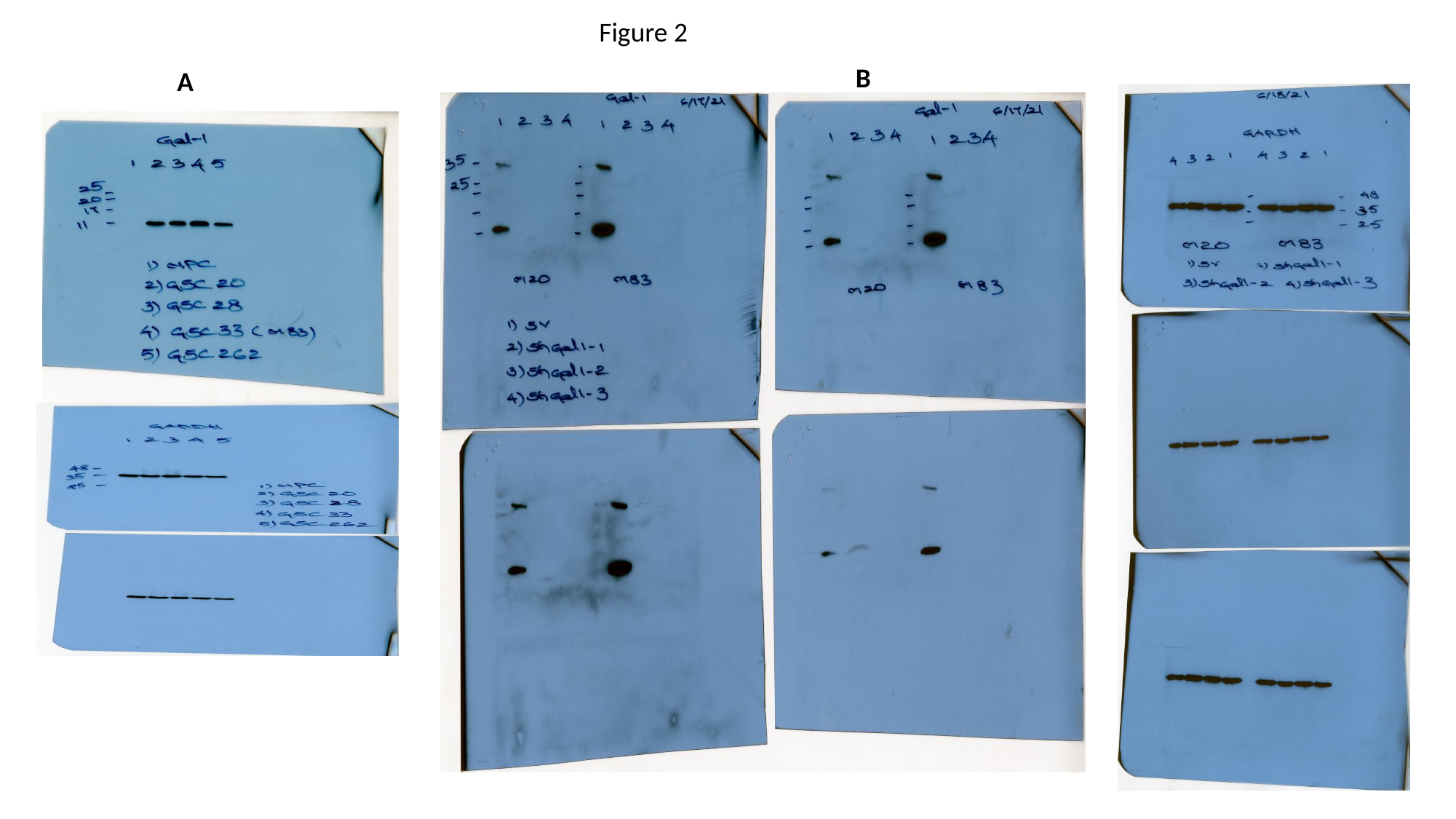

Figure 2
B
A

## Slide 3
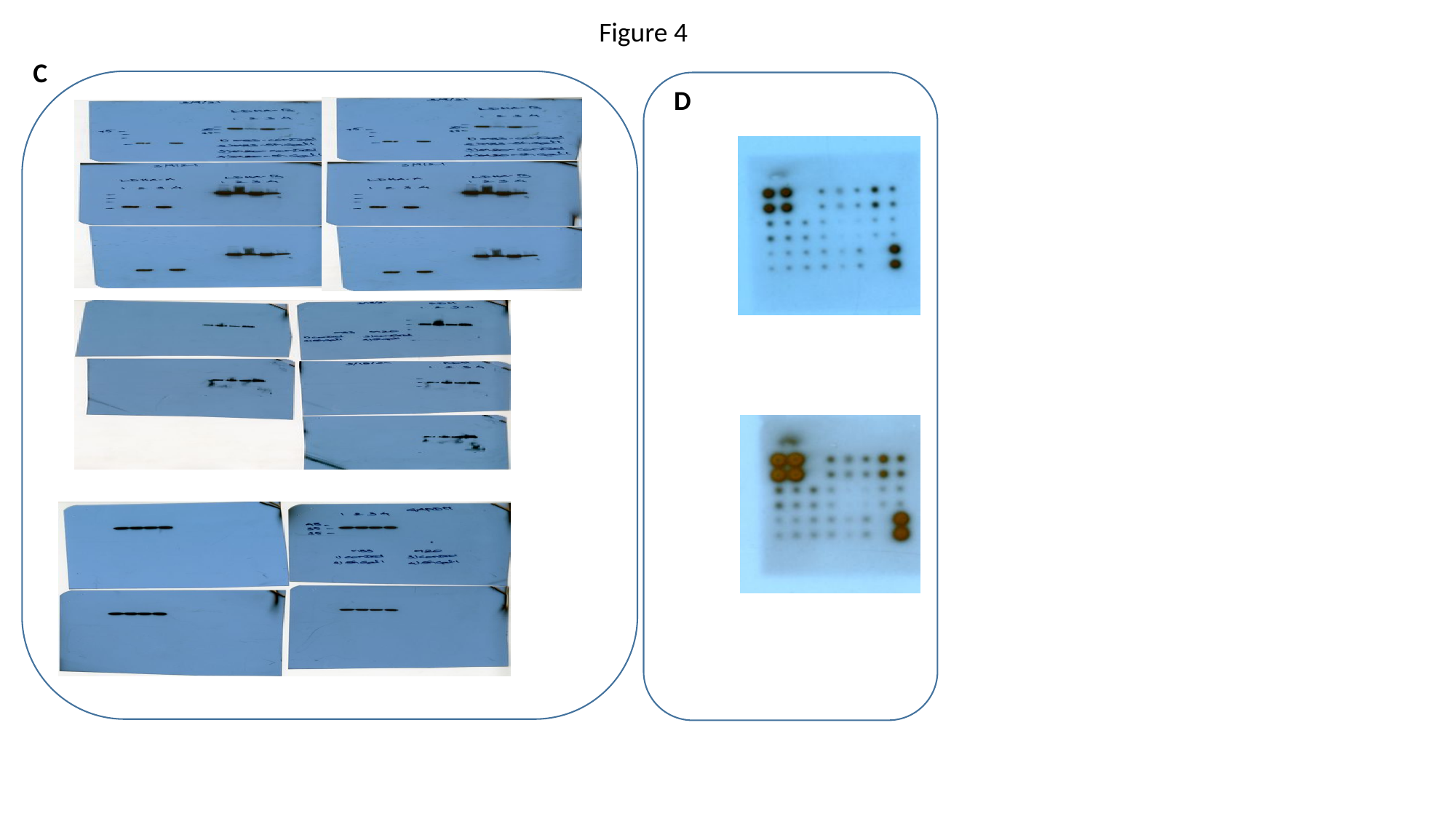

Figure 4
C
D

## Slide 4
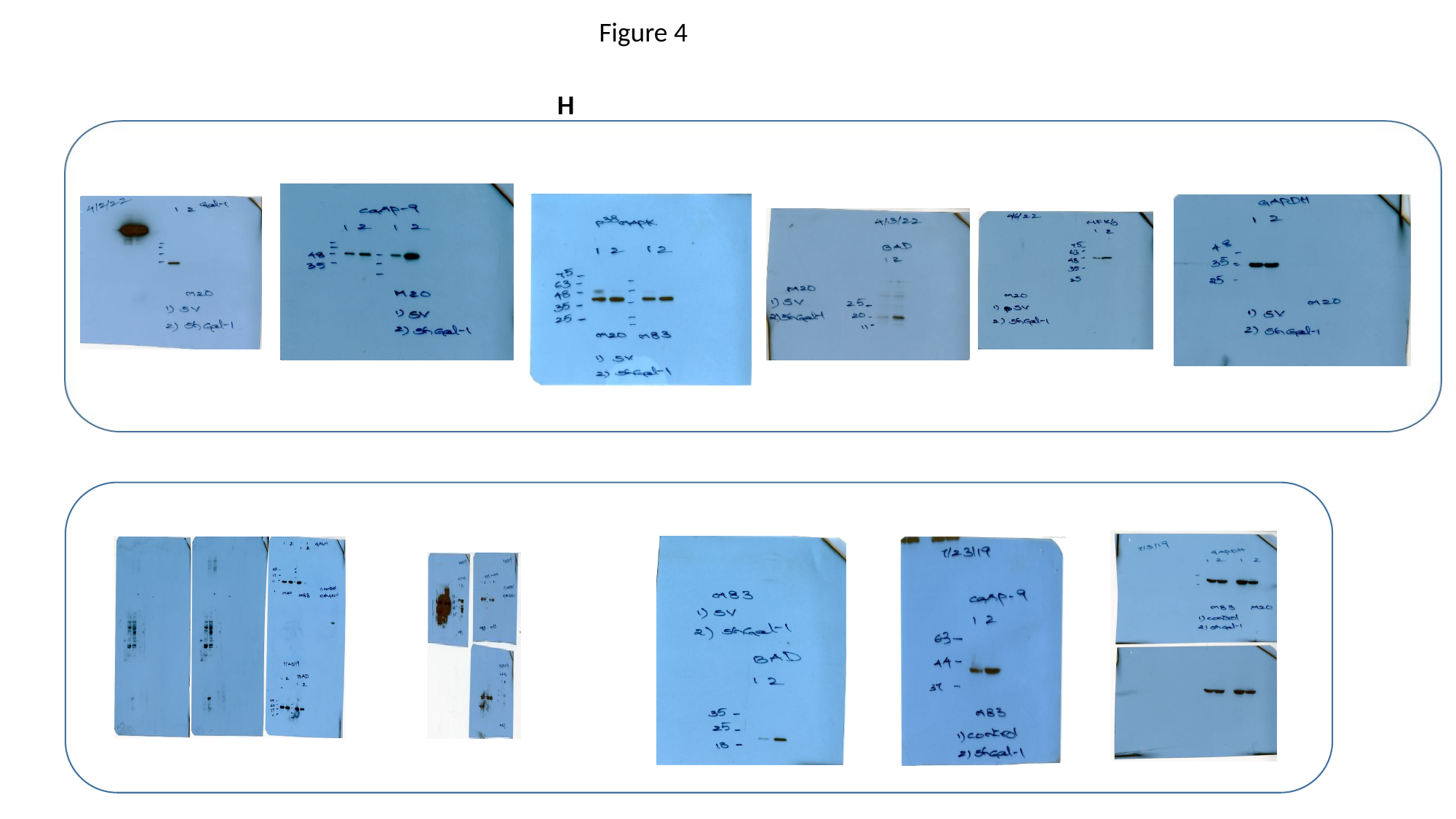

Figure 4
H

## Slide 5
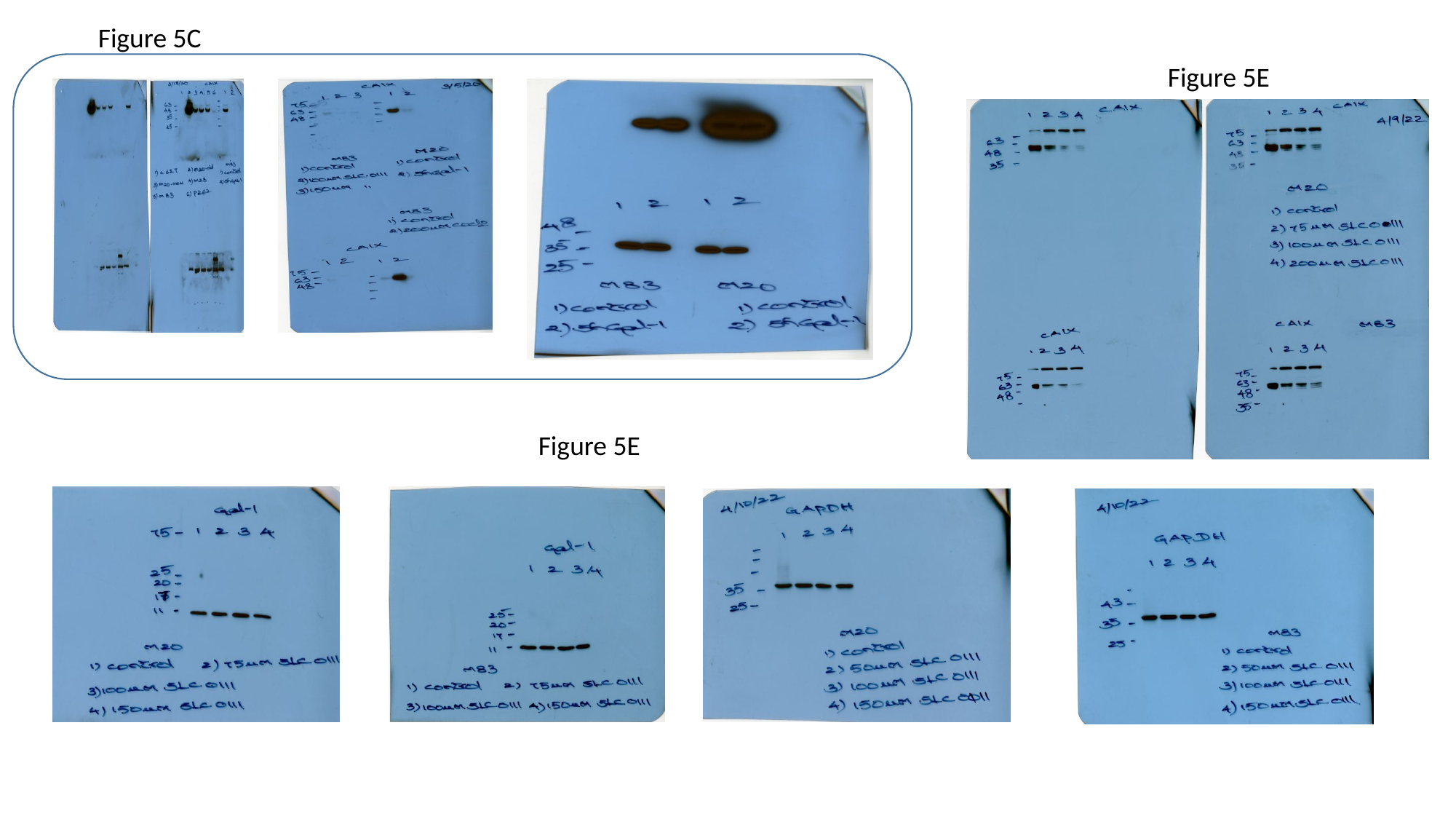

Figure 5C
Figure 5E
Figure 5E

## Slide 6
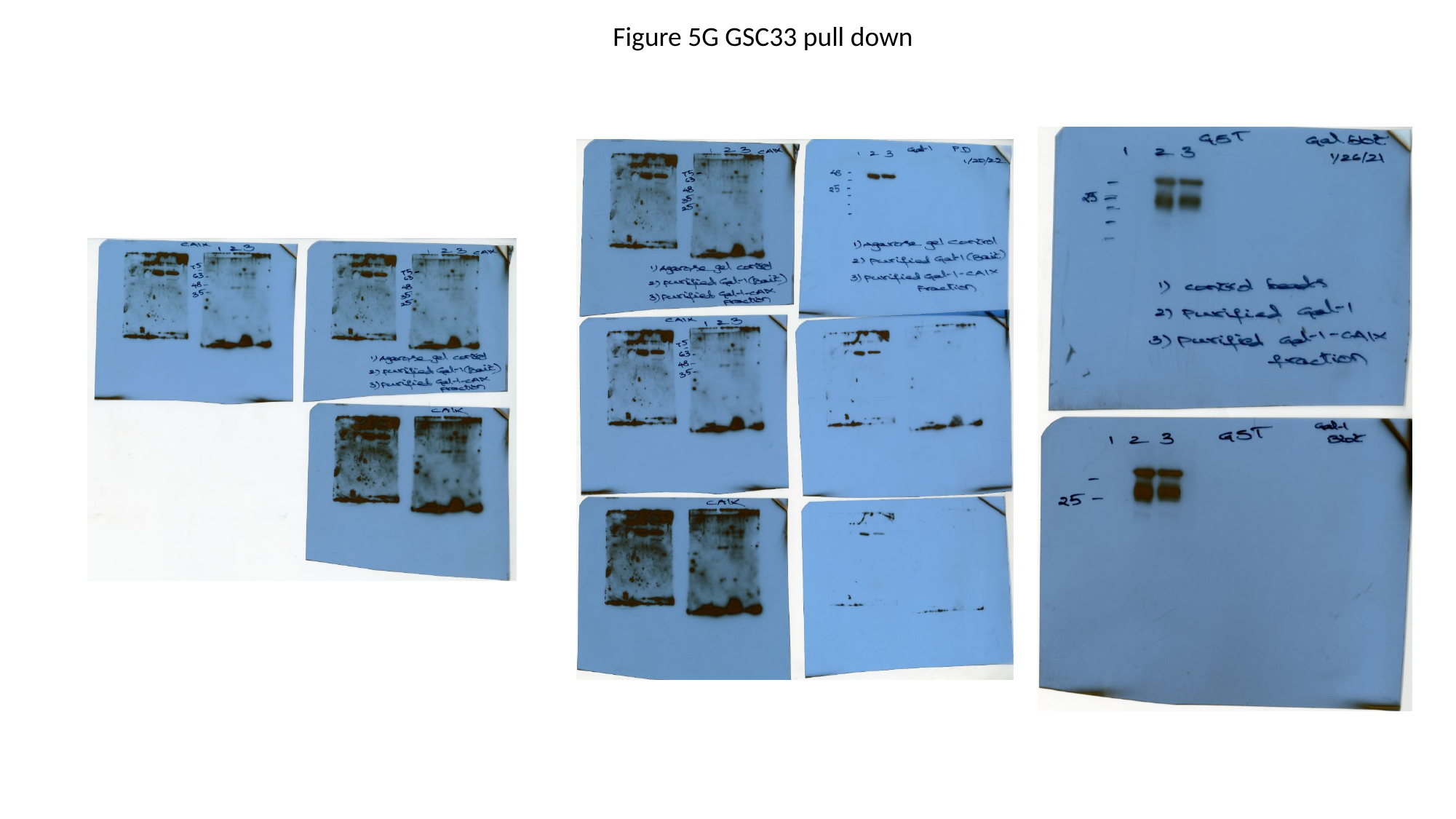

Figure 5G GSC33 pull down

## Slide 7
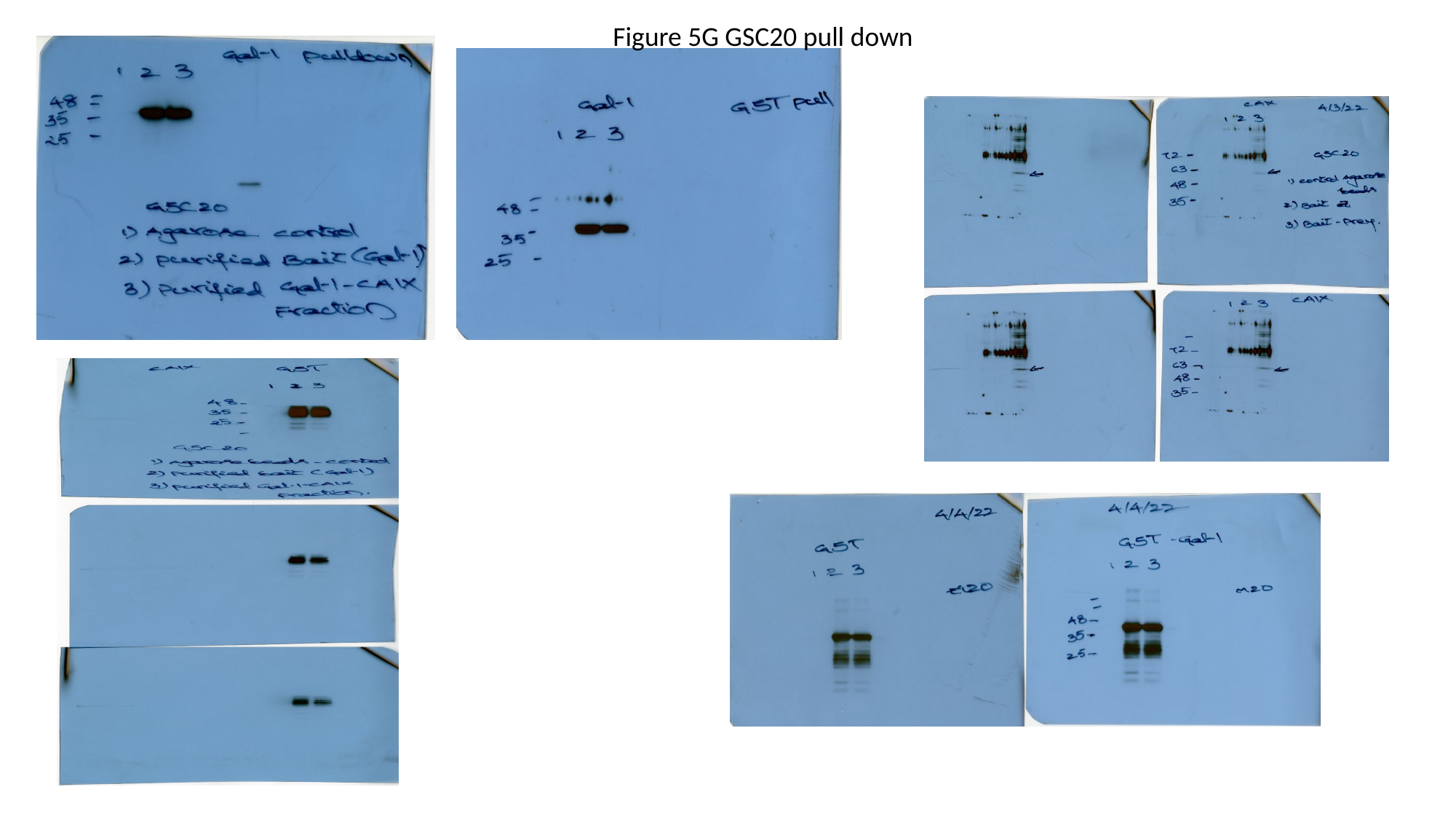

Figure 5G GSC20 pull down

## Slide 8
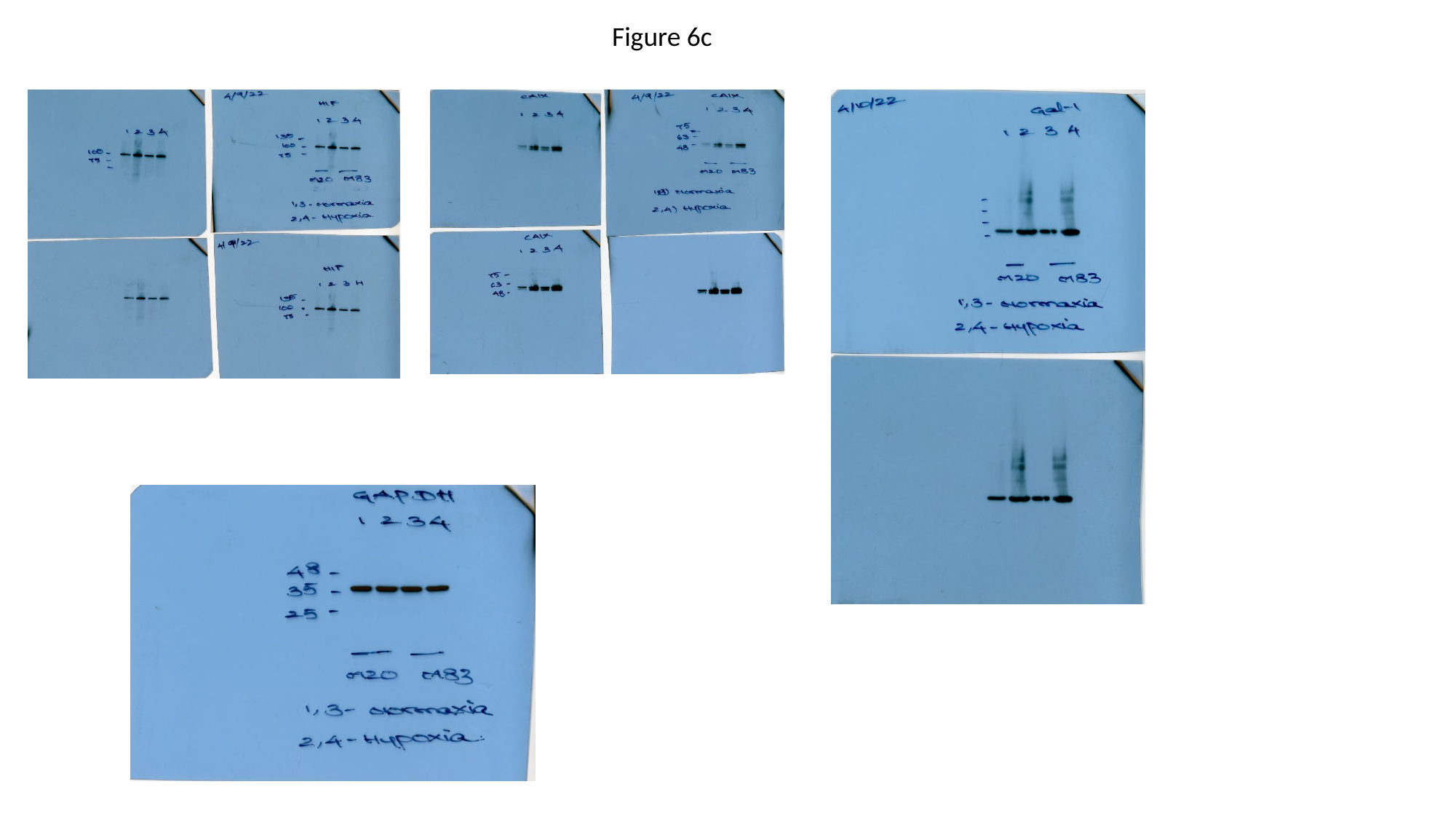

Figure 6c

## Slide 9
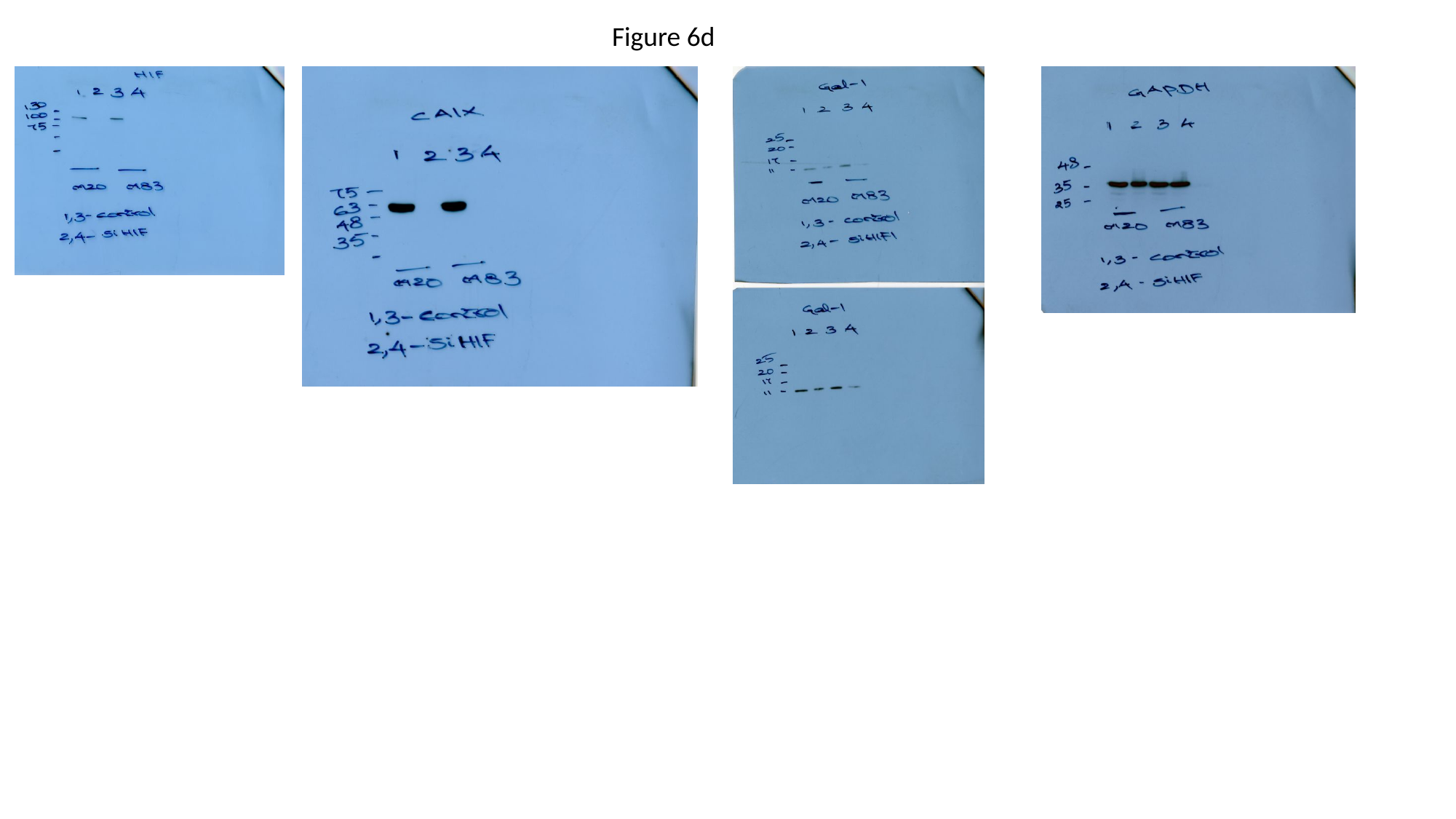

Figure 6d
